# Adsorption of Cu(II) on Oxidized Multi-Walled Carbon Nanotubes in the Presence of Hydroxylated and Carboxylated Fullerenes

**DOI:** 10.1371/journal.pone.0072475

**Published:** 2013-08-29

**Authors:** Jing Wang, Zhan Li, Shicheng Li, Wei Qi, Peng Liu, Fuqiang Liu, Yuanlv Ye, Liansheng Wu, Lei Wang, Wangsuo Wu

**Affiliations:** 1 Radiochemistry Laboratory, School of Nuclear Science and Technology, Lanzhou University, Lanzhou, PR China; 2 Institute of Modern Physics, Chinese Academy of Sciences, Lanzhou, PR China; 3 Institute of Nuclear Physics and Chemistry, China Academy of Engineering Physics, Mianyang, PR China; German Cancer Research Center, Germany

## Abstract

The adsorption of Cu(II) on oxidized multi-walled carbon nanotubes (oMWCNTs) as a function of contact time, pH, ionic strength, temperature, and hydroxylated fullerene (C_60_(OH)_n_) and carboxylated fullerene (C_60_(C(COOH)_2_)_n_) were studied under ambient conditions using batch techniques. The results showed that the adsorption of Cu(II) had rapidly reached equilibrium and the kinetic process was well described by a pseudo-second-order rate model. Cu(II) adsorption on oMWCNTs was dependent on pH but independent of ionic strength. Compared with the Freundlich model, the Langmuir model was more suitable for analyzing the adsorption isotherms. The thermodynamic parameters calculated from temperature-dependent adsorption isotherms suggested that Cu(II) adsorption on oMWCNTs was spontaneous and endothermic. The effect of C_60_(OH)_n_ on Cu(II) adsorption of oMWCNTs was not significant at low C_60_(OH)_n_ concentration, whereas a negative effect was observed at higher concentration. The adsorption of Cu(II) on oMWCNTs was enhanced with increasing pH values at pH < 5, but decreased at pH ≥ 5. The presence of C_60_(C(COOH)_2_)_n_ inhibited the adsorption of Cu(II) onto oMWCNTs at pH 4–6. The double sorption site model was applied to simulate the adsorption isotherms of Cu(II) in the presence of C_60_(OH)_n_ and fitted the experimental data well.

## Introduction

Carbon-based nanomaterials have been widely studied [1] as a new material with special mechanical, electrical, optical, catalytic, magnetic and photon sensitive properties. Applications for environmental pollution and biological toxicity problems should not be ignored [2], [3] considering the increasing utilization of carbon-based nanomaterials in industry, agriculture, and biology.

Given their large specific surface area which can be easily functionalized, carbon nanotubes are widely used as adsorbents for removal of various heavy metal ions from aqueous solutions [4], [5]. Cu(II) is a typical bivalent heavy metal pollutant which has been studied with respect to the uptake of Cu(II) onto various CNTs from water [6]–[13]. Chao-Yin [7] studied aqueous Cu^2+^ adsorption onto as-grown and modified carbon nanotubes and revealed that the modification of carbon nanotubes with H_2_SO_4_/KMnO_4_ increased not only the area of active adsorption sites on carbon nanotubes but also the proportion of available adsorption sites. Pyrzyńska and Bystrzejewski [8] reported that the adsorption capacity of carbon nanotubes and carbon-encapsulated magnetic nanoparticles for Cu(II) and Co(II) was greater than that of activated carbons. They deduced the primary reasons were surface charge densities, particle size effects at high ionic strengths, and overall degree of graphitization. Gao et al. [9] studied the removal of nickel, copper, zinc, and cadmium from multi-component solutions by the oxidized multi-walled carbon nanotubes indicating that the significant factors are surface features, ion exchange, and electrochemical potential. From the literature, it is concluded that carbon nanotubes possess strong adsorption affinity for heavy metal ions. Once discharged into the environment as pollutants, carbon nanotubes inevitably are combined with heavy metal pollutants or toxic organic macromolecules through adsorption, which leads to more complicated environmental pollution problems [14], [15].

In order to simulate the real effect between carbon nanotubes and heavy metal ions in the real environment, organic matter is often evaluated as one of the factors frequently studied due to its enhancement of Cu(II) adsorption onto carbon nanotubes. Organic compounds such as humic acid (HA) and fulvic acid (FA) are common in soil [16]. Noxious aromatic compounds such as the herbicide atrazine [17] and chemical materials such as acetone [18] widely found in the chemical industry. Sheng et al. [19] found that the adsorption of Cu(II) to multi-walled carbon nanotubes (MWCNTs) is significantly influenced by HA/FA and other organics. Tan et al. [20] reported counterion effects of nickel and sodium dodecylbenzene sulfonate (SDBS) adsorption to MWCNTs surfaces plays an important role in the nickel adsorption process.

Almost all of the researchers select a single kind of carbon nanomaterial as an adsorbent. However, very few researchers have reported the effect of the adsorption of metal ions on carbon nanotubes accompanied by another species of carbon nanomaterials. In fact, carbon nanomaterials as contaminants are not single structures in the environment but are an admixture of nanometer material with different sizes, structures and properties [21]–[23]. Therefore, it is necessary to investigate the adsorption mechanism of metal ions together with various combinations of carbon nanomaterials. It is difficult to achieve complete separation in the presence of a several species of carbon nanomaterials, so distinguishing the contribution of each carbon nanomaterial to the adsorption of metal ions is extremely difficult. Thus, we studied the three component system comprised of Cu(II) and carbon nanotubes in the presence of soluble fullerenes to evaluate the multi-component interactions.

Fullerenes (C_60_) are nanometer carbon materials which were widely studied prior to carbon nanotubes [24], [25]. Due to special physical, optical and superconducting performance, C_60_ has promising applications for gas storage, sensors, reinforced metals, superconducting, catalysts, cancer therapy, and other medical applications [26], [27]. With its well defined structure and π electron character [24], C_60_ readily forms π-π stacking interactions with other aromatic materials [28], [29]. These combined reactants then affect the adsorption of metal ions or organic matter on the composite material. Therefore, research on the three component system soluble fullerene plus Cu(II) and carbon nanotubes provides a reference for studying the other reactions between carbon nanomaterials and metal ions in various natural conditions. The study of the three component system also provides a theoretical basis for studying biological safety problems caused by multicomponent environment pollution.

## Experimental Process

### Materials

MWCNTs (prepared by chemical vaporization deposition ) were purchased from Shenzhen Nanotech Port Co., Ltd. China, with an outer diameter range of 10−30 nm, 1−10 μm in length, and a purity above 96% (including amorphous carbon <3%, ash <0.2%). Oxidized MWCNTs were prepared by oxidization with concentrated nitric acid-sulfuric acid (1:3, V/V). The as-prepared MWCNTs were immersed in a 500 mL flask containing 400 mL concentrated nitric acid and refluxed at 80°C for 24 h, and then washed by deionized water. After cleaning, the MWCNTs were re-dispersed in a mixture of 400 mL concentrated nitric acid and concentrated sulfuric acid (1∶3, V/V)and refluxed for 48 h at 80°C. Finally, the treated MWCNTs were filtered and washed to obtain oxidized MWCNTs (oMWNTs).

Fullerenes (C_60_), purity >99%, were purchased from Henan Puyang Yongxin Fullerene Technology Co., LTD. C_60_(OH)_n_ (n = 2∼24) was synthesized as given in a reported procedure [30]. 50 mg C_60_ was dissolved in 50 mL benzene, followed by additions of 2 mL of 2 mol/L NaOH, 5 drops of Tetra-Butyl-Ammonium Hydroxide (TBAH, 40% in water), and 5 mL 30% H_2_O_2_. This mixture was stirred at room temperature for 2 hours. After removal of benzene by separatory funnel, 20 mL methanol was added to produce a yellowish brown precipitate which was dissolved in aqueous solution. The above procedures were repeated 4∼5 times in order to completely remove TBAH and NaOH. Drying the precipitate under reduced pressure below 50°C yielded a yellow brown product. The sample was then dissolved in water, laid aside overnight, and then methanol was added to produce a precipitate. The fullerol was thus obtained by drying the precipitate under reduced pressure below 50°C. Synthesis of C_60_ water soluble di-malonic acid derivative C_60_(C(COOH)_2_)_n_ has been described in the literature [31]. After NaH was added to the solution of C_60_ in toluene, the solution changed from purple to dark red. Next diethyl bromomalonate was added. After vacuum and stirring under argon protection for 10h, the solution was filtered in order to remove the precipitate, and the solvent was removed in vacuum. The residue was eluted by toluene, and then 20 fold excess NaH was added. The solution was stirred under argon at 80°C for 10 h. After the heating source was removed, methanol was added for reaction termination followed by addition of HCl. The solution was cooled in air. The precipitate was collected by centrifugation, and thus washed with methanol, HCl, and deionized water and repeated twice. Finally, the resulting filtrate was dried in vacuum. The brown dried powder was C_60_(C(COOH)_2_)_n_.

All chemicals were of analytical reagent grade and all solutions were prepared with deionized water.

### Characterization

The changes in morphology and features of oMWCNTs before and after Cu(II) adsorption in the presence of two species of soluble fullerene were characterized by transmission electron microscope (TEM, Tecnai^TM^ G^2^ F30, FEI, US). The specific surface area of the oMWCNTs was measured using the Brunauer–Emmett–Teller nitrogen physisorption method (N_2_-BET), using a Surface Area and Porosity Analyzer (ASAP 2020M, Micromeritics Instrument Corporation, USA). Fourier transform infrared Spectroscopy (FTIR, Nexus670, Thermo Nicolet, American) was used in the analysis of the chemical surface groups of oMWCNTs. The resolution was 1 cm^−1^. The detector was DTGS KBr with working range between 400 and 4000 cm^−1^. The structural information of oMWCNTs was evaluated by a Raman spectroscopy (Jobin-Yvon LabRam HR80, HORIBA Ltd., France) with 514 nm laser. The zeta potential of oMWCNTs after C_60_(OH)_n_ and C_60_(C(COOH)_2_)_n_ adsorption were measured using a Zetasizer Nano ZS (Nano ZS ZEN3600, Malvern Instruments Ltd, Britain). The pH values of oMWCNTs solution were adjusted 6 by adding 0.1 M HCl to the glass beaker at 298K. For the FTIR and N_2_-BET analysis, the samples were equilibrated for 2 days and then centrifugation followed by washing with blank electrolyte to remove unbound soluble fullerene and Cu(II). The samples were then dried at 50°C.

### Adsorption experiments

The adsorption of Cu(II) on oMWCNTs was investigated by using a batch technique in a 10 mL polyethylene centrifuge tube. The stock solutions of oMWCNTs and NaCl were pre-equilibrated for 24 h and then Cu(II) stock solution was added to achieve the desired concentrations. The system was adjusted to the desired pH by adding negligible volumes of 0.01 or 0.1 mol·L^−1^ HCl or NaOH. After the suspensions were equilibrated for 2 days (which was enough to achieve equilibrium), the solid and liquid phases were separated by using centrifugation at 12,000 rpm for 30 min. The residual Cu(II) concentrations were determined at 540 nm with UV-VIS spectrophotometry (Perkin-Elmer, American). The adsorbed quantity of Cu(II) was calculated from the difference between the initial concentration and that at equilibrium.

Adsorption isotherms were developed using the batch technique in polyethylene centrifuge tubes under ambient conditions at 293, 313, and 333 K, respectively. The stock solutions of 0.01 mol/L NaCl and 0.5 g/L oMWCNTs were pre-equilibrated for 24 h before addition of Cu(II) stock solution. The initial concentrations of Cu(II) were from 1.0 to 20.0 mg/L.

The effects of C_60_(OH)_n_/C_60_(C(COOH)_2_)_n_ concentration on Cu(II) adsorption was also investigated. The stock solutions of oMWCNTs, C_60_(OH)_n_/C_60_(C(COOH)_2_)_n_, and NaCl were pre-equilibrated for 24 h before adding the Cu(II) stock solution. The experimental sequences were as follows: (1) oMWCNTs, C_60_(OH)_n_/C_60_(C(COOH)_2_)_n_ and NaCl were pre-equilibrated before the addition of Cu(II) (defined as (oMWCNTs+C_60_(OH)_n_/C_60_(C(COOH)_2_)_n_)+Cu); (2) oMWCNTs, Cu(II) and NaCl were pre-equilibrated before the addition of C_60_(OH)_n_/C_60_(C(COOH)_2_)_n_ (defined as (oMWCNTs+Cu)+C_60_(OH)_n_/C_60_(C(COOH)_2_)_n_); (3) C_60_(OH)_n_/C_60_(C(COOH)_2_)_n_, Cu(II) and NaCl were pre-equilibrated before the addition of oMWCNTs (defined as (C_60_(OH)_n_/C_60_(C(COOH)_2_)_n_+Cu)+oMWCNTs).

## Results and Discussion

### Characterization of purified oMWCNTs, C_60_(OH)_n_ and C_60_(C(COOH)_2_)_n_

**Figure 1:**
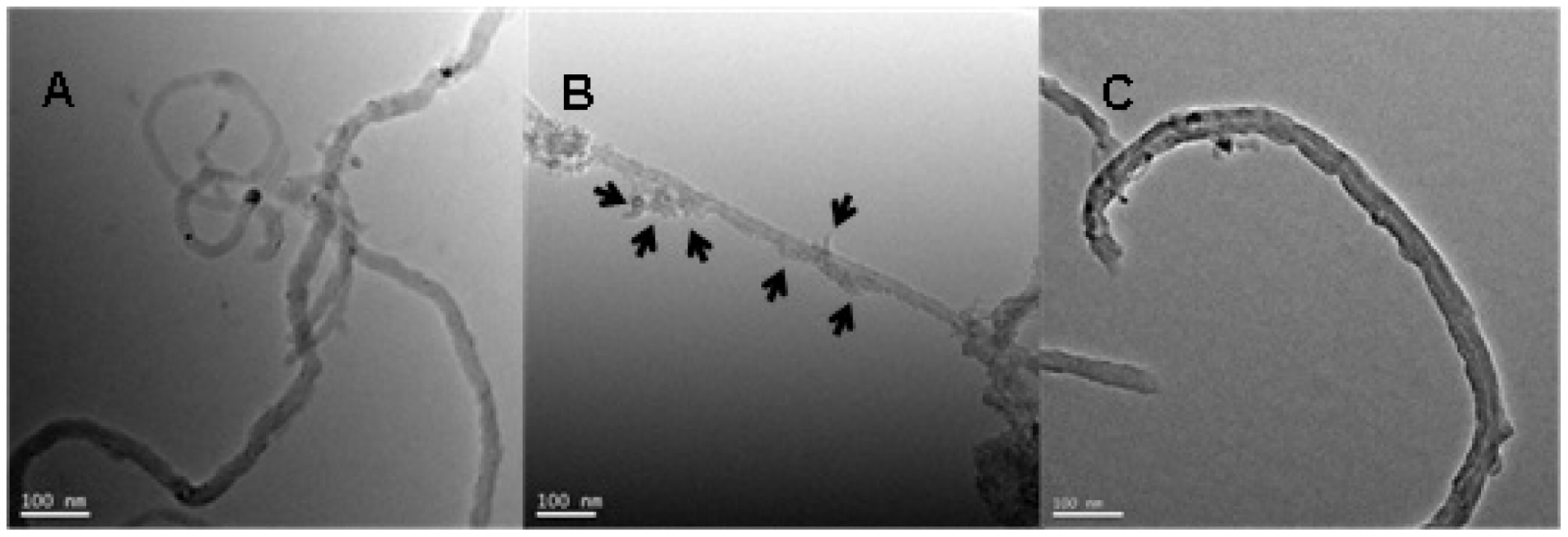
TEM photographs of (A) oMWCNTs; (B) oMWCNTs+C_60_(OH)_n_; (C) oMWCNTs +C_60_(C(COOH)_2_)_n_.

Figure 1 shows TEM photomicrographs of oMWCNTs, C_60_(OH)_n_-oMWCNTs dispersion, and C_60_(C(COOH)_2_)_n_-oMWCNTs dispersion in aqueous solution, respectively. Carbon nanotubes (Figure 1A) have an integrated hollow tubular structure after oxidation, indicating that their surfaces are smooth, without any obvious amorphous carbon and other particles on their walls. For the sample of C_60_(OH)_n_-oMWCNTs dispersion (Figure 1B), some particles (seen the represent of arrow in Figure 1B) persisted on the walls of CNTs. It can be discerned that C_60_(OH)_n_ particles are heterogeneously attached to the surface of oMWCNTs. As can be seen from Figure 1C, the surface of oMWCNTs are not coated with C_60_(C(COOH)_2_)_n_. This may be attributed to differences in functional substitution on the surface of C_60_.

The surface area, mesopore volumes, and pore size (Table 1) are 100 m^2^/g, 0.34 cm^3^/g, and 13.326 nm, respectively.

**Table 1:** Characteristics of the porous structure of the oMWCNTs.

Sample	S_BET_(m^2^/g)	Total pore volumes (cm^3^/g)	Pore size (nm)
oMWCNTs	100	0.34	13.326

**Figure 2:**
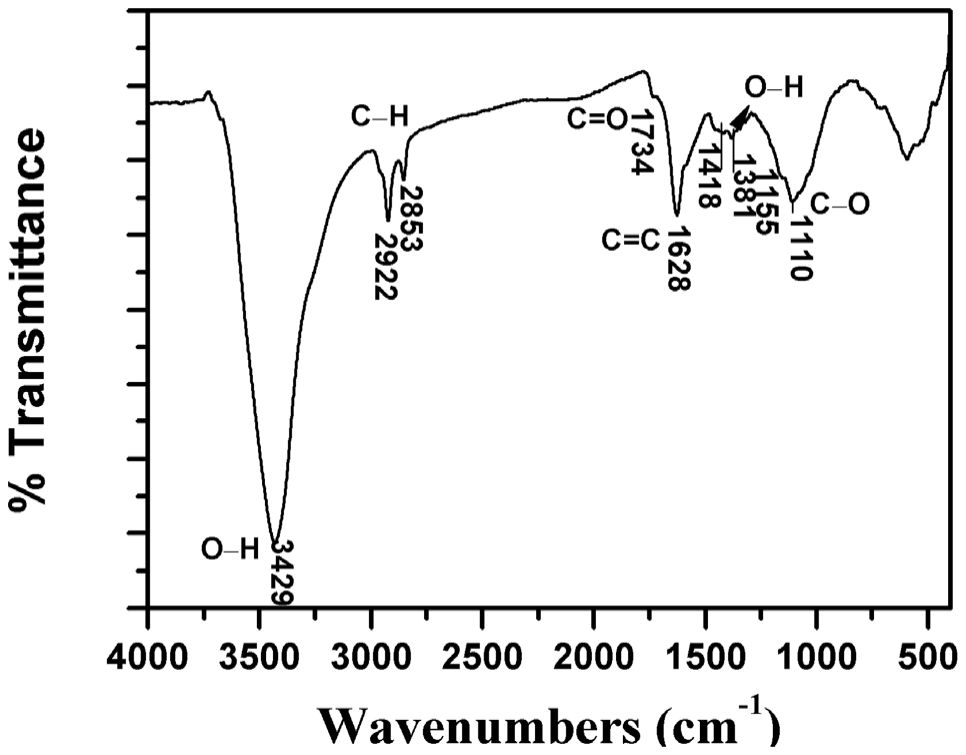
FTIR spectrum of oMWCNTs.

Figure 2 presents the FTIR spectra of modified CNTs. The peak at 3429 cm^−1^ can be assigned to −OH stretching vibration mode of carboxylic groups (−COOH and −COH), while the peaks at 2922 and 2853 cm^−1^ can be related to the asymmetric and symmetric −CH stretching vibration mode of the sidewalls, the peak at 1734 cm^−1^ can be attributed to stretching vibrations of carbonyl groups (C = O) present in carboxylic groups (−COOH). The spectra of conjugated C = C stretching bands appeared at about 1628 and 1418 cm^−1^, the peak at 1561 cm^−1^ is associated with the carboxylic and carboxylate anion stretching mode, the peak between 1000 and 1380 cm^−1^ can be attributed to C−O stretching and −OH bending modes of alcoholic, phenolic and carboxylic groups [32], [33].

**Figure 3:**
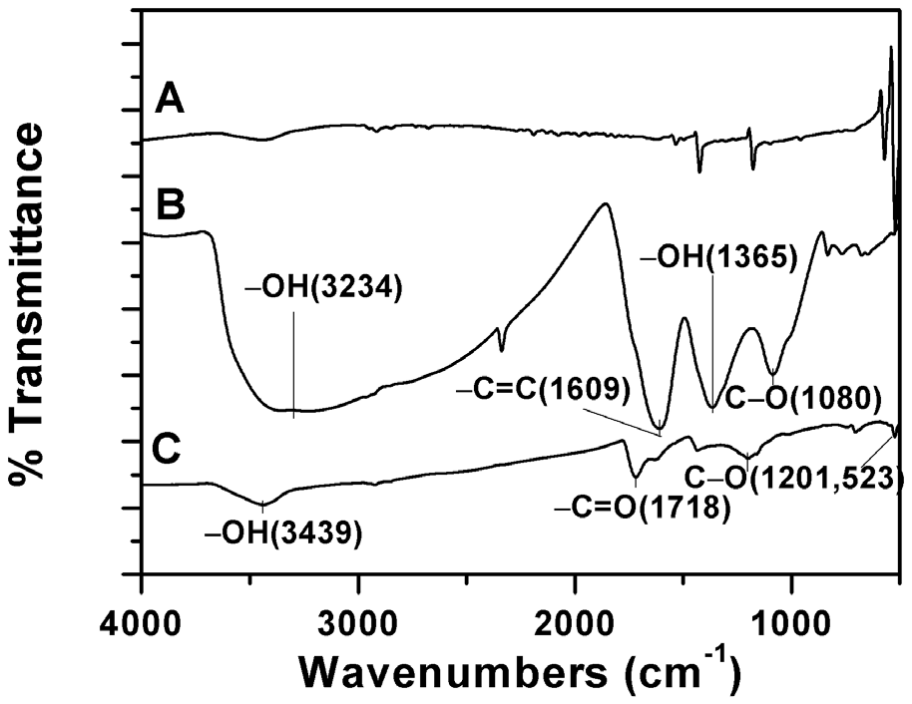
FTIR spectrum of (A) raw C_60_; (B) C_60_(OH)_n_; (C) C_60_(C(COOH)_2_)_n_.

Figure 3 shows the FTIR spectrum of raw C_60_, C_60_(OH)_n_ and C_60_(C(COOH)_2_)_n_, respectively. For C_60_(OH)_n_ (Figure 3B), the FTIR shows a broad hydroxyl adsorption centered at 3234 cm^−1^, the peak at 1609 cm^−1^ can be assigned to the C = C stretching vibration mode. The peak at 1086 and 1365 cm^−1^ are related to C−O stretching vibration mode and −OH in-plane bending vibration mode. These peaks are all the infrared characteristic peaks of C_60_(OH)_n_ [34]. The FTIR spectrum of C_60_(C(COOH)_2_)_n_ (Figure 3C) exhibits main peaks at 3439, 1718, 1201 and 523 cm^−1^. A previous report [35] suggested that hydroxyl (−OH), carbonyl (>C = O) and carboxyl (−C−O) were present on the surfaces of C_60_(C(COOH)_2_)_n_.

FTIR is also conducted on oMWCNTs after C_60_(OH)_n_/C_60_(C(COOH)_2_)_n_ adsorption and their corresponding spectra are shown in Figure S1 in File S1. It can be seen that after adsorption, the same infrared absorptions still remained. C_60_(OH)_n_ adsorbed on oMWCNTs (Figure C in File S1) exhibit there are increase in intensity of same bands at 2922 and 2853 cm^−1^ (assigned to asymmetric and symmetric CH_2_ stretching), 1734 cm^−1^ (assigned to C = O stretching vibrations), 1628 and 1418 cm^−1^ (assigned to C−O stretching). The new peak at 1404 cm^−1^ can be assigned to aliphatic hydroxyl bending. These changes may be result from C_60_(OH)_n_ adsorbed on the surface of oMWCNTs [32].

**Figure 4:**
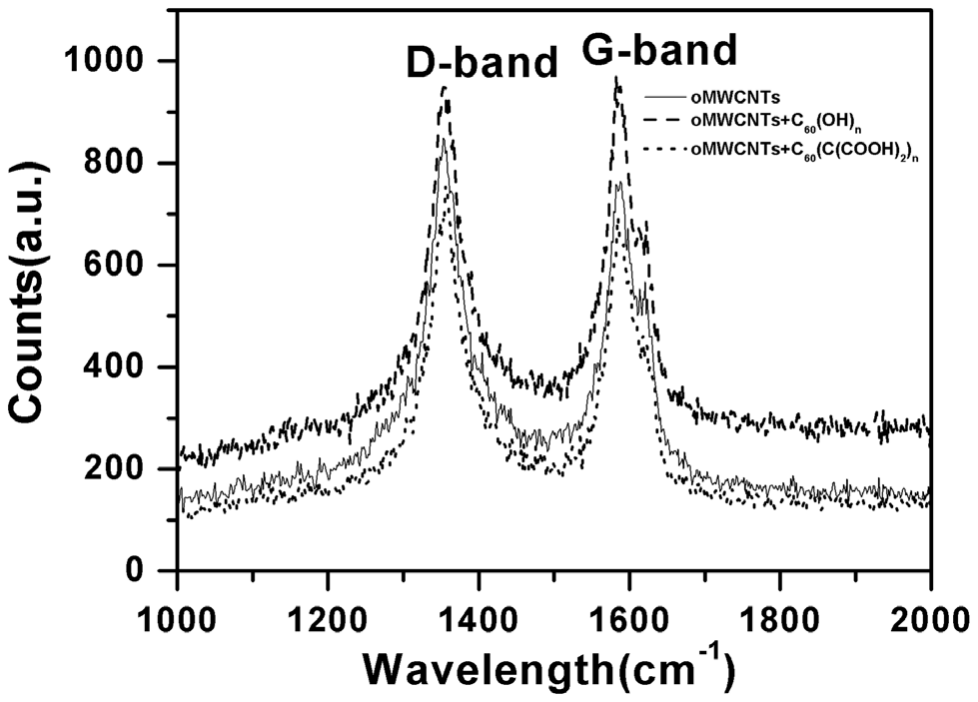
Raman spectra of oMWCNTs before and after C_60_(OH)_n_/C_60_(C(COOH)_2_)_n_ adsorption.

Raman spectroscopy of oMWCNTs presents in Figure 4 are composed of two characteristic peaks. The peak near 1350 cm^−1^ is the D-band corresponding to the disordered sp^2^-hybridized carbon atoms of nanotubes while the peak near 1580 cm^−1^ is the G-band corresponding to the structural integrity of sp^2^-hybridized carbon atoms of nanotubes [19]. As can be observed, the G-band of oMWCNTs shows an increase after C_60_(OH)_n_ adsorption. The G band is due to the bond stretching of both aromatic and aliphatic C−C pairs [36]–[38]. This suggests that oMWCNTs exit more crystalline graphitic structures after the adsorption C_60_(OH)_n_. Figure S3 and Figure S4 in File S1 plot the zeta potential of oMWCNTs. All of zeta potentials of oMWCNTs become more negative as the concentration of C_60_(OH)_n_/C_60_(C(COOH)_2_)_n_ increased. Meanwhile the change of oMWCNTs zeta potential with increasing of C_60_(OH)_n_ concentration is more regular than that with increasing of C_60_(C(COOH)_2_)_n_ concentration. This demonstrates that the zeta potential of oMWCNTs have been changed by C_60_(OH)_n_ adsorbed. Figure S5 in File S1 shows photographs of oMWCNTs and C_60_(OH)_n_/C_60_(C(COOH)_2_)_n_ solutions after centrifugation. The solid and liquid phases are separated by using centrifugation for samples “A” to “G”. Interestingly, it is noticed that somewhat better dispersion is achieved for sample “G”, which is consistent with the analysis of the oMWCNTs zeta potential (seen in Figure S3 and Figure S4 in File S1). Therefore, it is clear that the presence of C_60_(OH)_n_ can promotes oMWCNTs dispersion in adsorption system.

### Adsorption of Cu(II) on the oMWNTs

**Figure 5:**
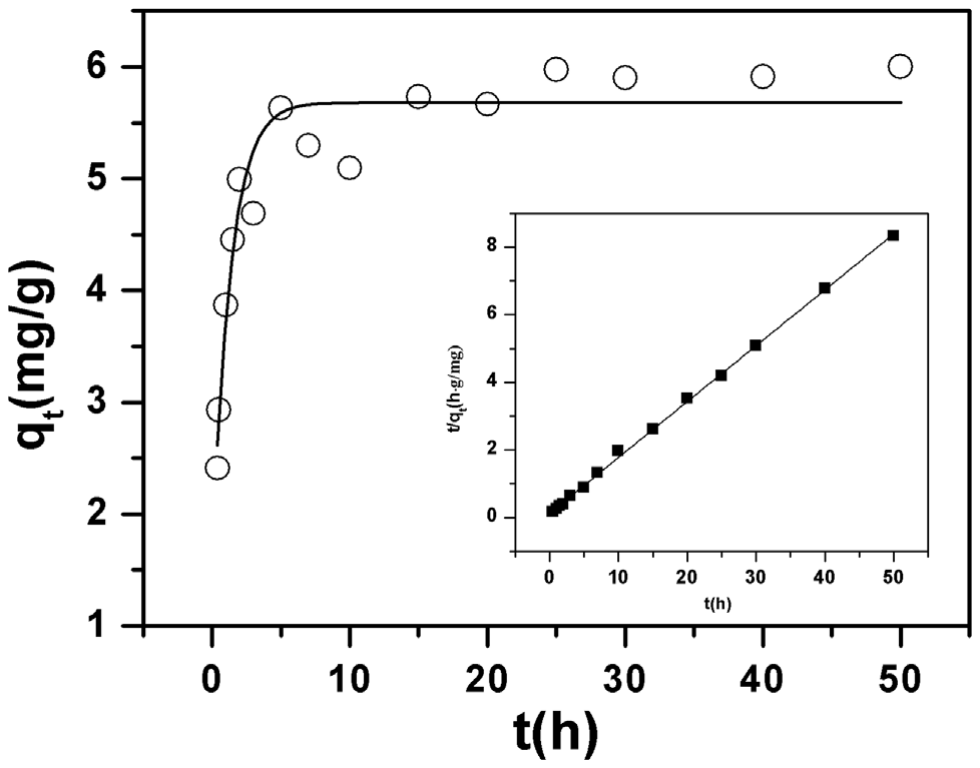
Effect of contact time on Cu(II) adsorption rate onto oMWCNTs and test of pseudo-second-order adsorption kinetics plot for Cu(II), *m/V*  = 0.5 g/L, *T = *25±1°C, C[Cu^2+^]initial  = 1.87×10^−4^ mol/L, *I* = 0.01 mol/L NaCl, pH = 4.00±0.05.



Figure 5 shows the effect of contact time on the adsorption capacity of Cu(II) on the surface of oMWNTs and the initial concentration of Cu(II) is 12 mg/L. As can be seen, the adsorption of Cu(II) onto oMWCNTs increases very quickly at the initial contact, then the adsorption remains at a steady state with increasing time after 5 h. The adsorption of Cu(II) mainly occurs with the surface functional groups of oMWCNTs. The ability of oMWCNTs to remove metal ions from aqueous solution is enhanced due to the strong interaction between Cu(II) and functional groups of oMWCNTs. The markedly increased transport rate of Cu(II) on the surface of oMWCNTs, and the adsorption capacity becomes stable after about 5 h. This adsorption process is generally similar the process of heavy metal ion adsorption on oMWCNTs [39]. It is well known that oxidation of the carbon surface could offer not only more hydrophilic surface structure but also a large number of oxygen-containing functional groups like −COOH, −OH, or −C = O on the surfaces of oMWCNTs, which increase the adsorption capability of the carbon material [40]. The pseudo-second-order rate equation (Eq. 1) [41] was employed to fit the adsorption data. Its general form is:

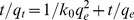
(1)where *q_t_* (mg/g) is the amount of Cu(II) adsorbed on oMWCNTs at time *t* and *q_e_* (mg/g) is the equilibrium adsorption capacity; *k_o_* (g/mg·h^−1^) is the pseudo-second-order rate constant of adsorption. The straight-line plots of *t/qt* vs. *t* (Figure 5) indicates that the kinetic adsorption of Cu(II) on oMWCNTs can be well described by the pseudo-second-order rate equation. The values of *k_o_* and *q_e_* are 1.28 g/mg·h^−1^ and 15.13 mg/g, respectively. These are calculated from the intercept and slope of Eq. (1). The correlation coefficient of the pseudo-second-order rate equation for the linear plot is 0.9999, which suggests that the kinetic adsorption can be very well described by the pseudo-second-order rate equation. The value of *k_o_* also indicates that the adsorption process rapidly achieves equilibrium [39]. Actually, the kinetic adsorption data have been simulated with first-order reversible model, pseudo-first-order model, pseudo-second-order model, and intraparticle diffusion model, respectively. The results have been listed in Table S3 in File S1. From the values of R^2^, the kinetic adsorption of Cu(II) can be fitted by the four models. However, pseudo-second-order model fitted the experimental data best among the four models. A similar results were also observed by Xu et al. [39] and Hu et al. [42]. According to the above results, the shaking time was fixed for 48 h for the rest of the batch experiments to assure that equilibrium was fully reached.

**Figure 6:**
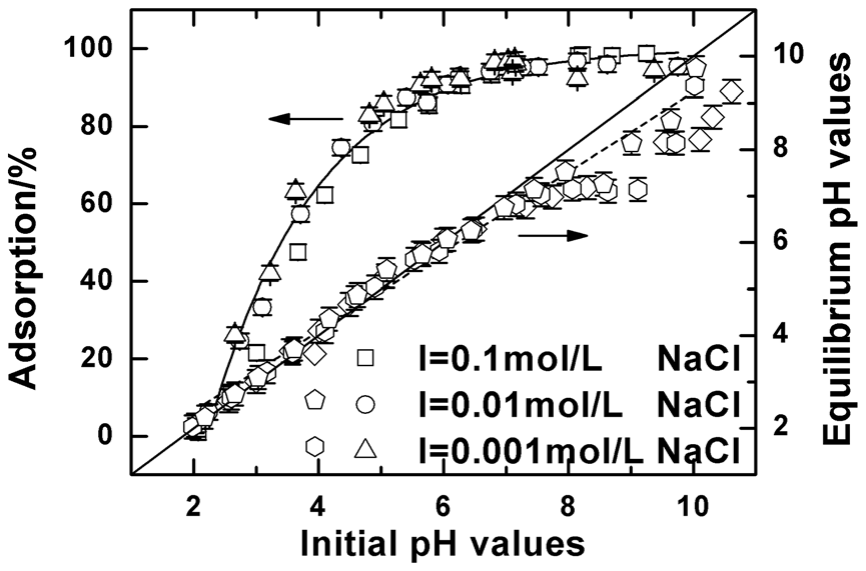
Adsorption of Cu(II) on oMWCNTs as a function of pH, *m/V*  = 0.5 g/L, *T = *25±1°C, C[Cu^2+^]initial  = 1.87×10^−4^ mol/L. Solid points: adsorption vs. initial pH values; open points: equilibrium pH values vs. initial pH values.

Solution pH is one of the important factors in controlling the adsorption experiments. Figure 6 shows the influence of pH from 2 to 10 on the adsorption of Cu(II) onto oMWCNTs in 0.001, 0.01 and 0.1 mol·L^–1^ NaCl solution. Cu(II) adsorption is strongly influenced by the system pH values. The adsorption percentage of Cu(II) increases sharply to about 96% at pH <6.5, and then maintains a high level state with pH >6.5. This is similar to the Cu(II) adsorption on the other materials [43]–[45]. Yang et al. [46] investigated the adsorption of Ni(II) on oMWCNTs and obtained similar results. They found that the adsorption of Ni(II) increased from 0% to 99% at pH 2–9, and then maintained the same high adsorption capacity with significantly increasing pH. The adsorption properties of oMWCNTs can be explained by the surface charge and the chemical properties of oMWCNTs [47]. The point of zero charge (pH_pzc_) of oMWCNTs is about 5 [19], [48]. In the aqueous system, the surface charge of oMWCNTs is positive at pH < pH_pzc_ because of protonation, whereas the surface becomes negative due to deprotonation at pH > pH_pzc_. With increasing pH, the sorption of Cu(II) increases due to the electrostatic attraction between the positive metal ions and the negative functional groups of oMWCNTs. Additionally, the distribution of Cu(II) species in solution is another important factor for pH dependent adsorption. Under the present experimental conditions, Cu(II)species can be present in the forms of Cu^2+^, Cu(OH)^+^, Cu_2_(OH)_2_^2+^, CuCO_3_(aq), Cu(CO_3_)_2_^2–^ at its initial concentration of 1.87×10^−4^ mol·L^−1^ in 0.01 mol·L^−1^ NaCl solution (as shown in Figure S2 in File S1). When pH is ≤6.5, the predominant species is Cu^2+^ and the extraction of Cu^2+^ is nearly accomplished. When pH >6.5, Cu(II) hydrolysis species (i.e. Cu(OH)^+^ and Cu_2_(OH)_2_^2+^) are the main form present in the solution. CuCO_3_(aq), and Cu(CO_3_)_2_^2−^ begin to occur due to the impact of CO_2_ at pH >7.5. These species have a relatively low solubility in aqueous phase, therefore Cu(II) adsorption on oMWCNTs maintains its maximum amount and remains stable.

The relationship between equilibrium pH value and initial pH is also reflected in Figure 6. The diagonal line represents that the value of pH in the adsorption process remains unchanged. However, the measured equilibrium pH values are slightly lower than the initial pH values, and decreases as the initial pH value increases. This is generally interpreted as the result of the −OH group (i.e. carboxyl and phenolic hydroxyl) being deprotonated on the surface of oMWCNTs. As the solution pH increases, more and more Cu^2+^ adsorbsto oMWCNTs surfaces and releases H^+^ into the solution, eventually leading to the decreasing equilibrium pH value in solution. Some researchers find that the pH value decreases with increasing initial metal ion concentration. This provides evidence that metal ion adsorption onto oMWCNTs surface causes H^+^ to be released from the functional groups into the solution, thus explaining the decline of pH in the solution [14], [49]. This explanation is consistent with our experimental results.

It can also be seen from Figure 6 that the background electrolyte concentrations have no significant effect on the adsorption of Cu (II) to oMWCNTs. This shows that Na^+^ in the solution is not involved in the adsorption of Cu (II) onto oMWCNTs. This is consistent with some of the results of adsorption of metal ions [50], [51] reported in the literature. The adsorption mechanism can be predicted by studying the change in ionic strength on the adsorption as proposed by Hayes and Leckie [52]. They believe that the adsorption reaction occurs mainly in the β plane when the ionic strength has a significant effect on the adsorption. They also conclude that the adsorption mechanism is predominately ion exchange. Conversely, complexation reactions may be occurring in the *o* plane without the influence of ionic strength. In our experiment, Cu(II) adsorption onto oMWCNTs predominately occurs in the *o* plane. Cu(II) adsorption on oMWCNTs is strongly dependent on pH, and largely independent of ionic strength, indicating that the adsorption mechanism is mainly chemical complexation rather than ion exchange.

**Figure 7:**
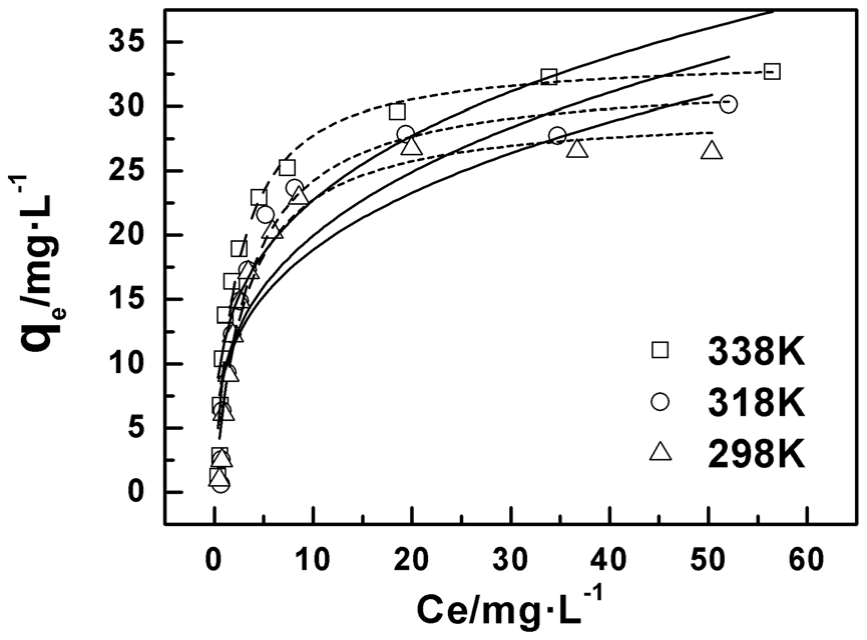
Adsorption isotherms of Cu(II) on oMWCNTs at three different temperatures, *m/V*  = 0.5 g/L, pH = 4.00±0.05, *I* = 0.01 mol/L NaCl, C[Cu^2+^]initial  = 1.87×10^−4^mol/L. Symbols denote experimental data, dotted lines represent the model fitting to the Langmuir equation, solid lines represent the model fitting to the Freundlich equation.



Figure 7 shows the isotherm for Cu(II) adsorption onto oMWCNTs. In order to describe the adsorption of Cu(II) on the oMWCNTs more accurately, the Langmuir and Freundlich models were employed to fit the experimental isotherm data. The general form of the Langmuir model is:

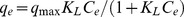
(2)Where *q_e_* (mg/g) and *c_e_* (mg/L) represents the concentration of the metal ions in the solid and liquid phases at equilibrium adsorption, respectively; *K_L_* (L/g) is the adsorption equilibrium constant; *q_max_* (mg/g) is the maximum adsorption capacity of the metal ion.

The general form of the Freundlich model is: 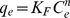
(3)Where *q_e_* (mg/g) represents the adsorption amount of the metal ions in the unit mass of oMWCNTs; *c_e_* (mg/L) is the equilibrium aqueous concentration; *K_F_* and *n* are the absorption capacity and strength in the Freundlich equilibrium constants, respectively.

The adsorption isotherm resulting from the two models above are shown in Figure 7. The obtained fitting parameters are listed in Table 2. The correlation coefficient of the Langmuir model is greater than that of Freundlich model. Therefore the Langmuir model fit better explains the Cu(II) adsorption data as compared with the Freundlich model. This is consistent with literature results that the adsorption of Cu(II) on the other materials best fit the Langmuir model [53], [54]. The results of Figure 7 also indicate that the adsorption capacity of oMWCNTs is larger when the temperature is higher, so the reaction is more likely to occur at higher temperatures. Moreover, the thermodynamic parameters of Δ*H*, Δ*G,* and Δ*S* obtained from different adsorption isotherms are shown in Table 3. As seen in Table 3, Δ*H* >0 indicates that the adsorption reaction is endothermic; Δ*G* <0, shows that the adsorption reaction is a spontaneous process. Therefore, high temperature is advantageous and promotes adsorption.

**Table 2:** The parameters of Langmuir and Freundlich fitting of Cu(II) adsorption on oMWCNTs.

	Langmuir constants			Freundlich constants		
*T*(K)	*q_max_*(mg·g^−1^)	*b*	R^2^	*K_F_*(mg^1−n^· L^n^·g^−1^)	*n*	R^2^
298	29.69	0.33	0.962	9.34	0.31	0.788
318	32.31	0.29	0.957	9.54	0.32	0.805
338	33.97	0.44	0.965	11.72	0.29	0.824

**Table 3:** Values of thermodynamic parameters for the adsorption of Cu(II) onto oMWCNTs.

C_0_(mg·L^−1^)	ΔH^0^(KJ·mol^−1^)	ΔS^0^(J·mol^−1^·K^−1^)	ΔG^0^(KJ·mol^−1^)		
			298K	318K	338K
4	11.83	112.19	−21.6	−23.85	−26.09
12	8.05	94.68	−20.16	−22.06	−23.95
16	5.11	82.63	−19.51	−21.17	−22.82
20	3.62	71.96	−17.82	−19.26	−20.7

### Influence of C_60_(OH)_n_ on the adsorption of Cu(II) onto oMWCNTs

**Figure 8:**
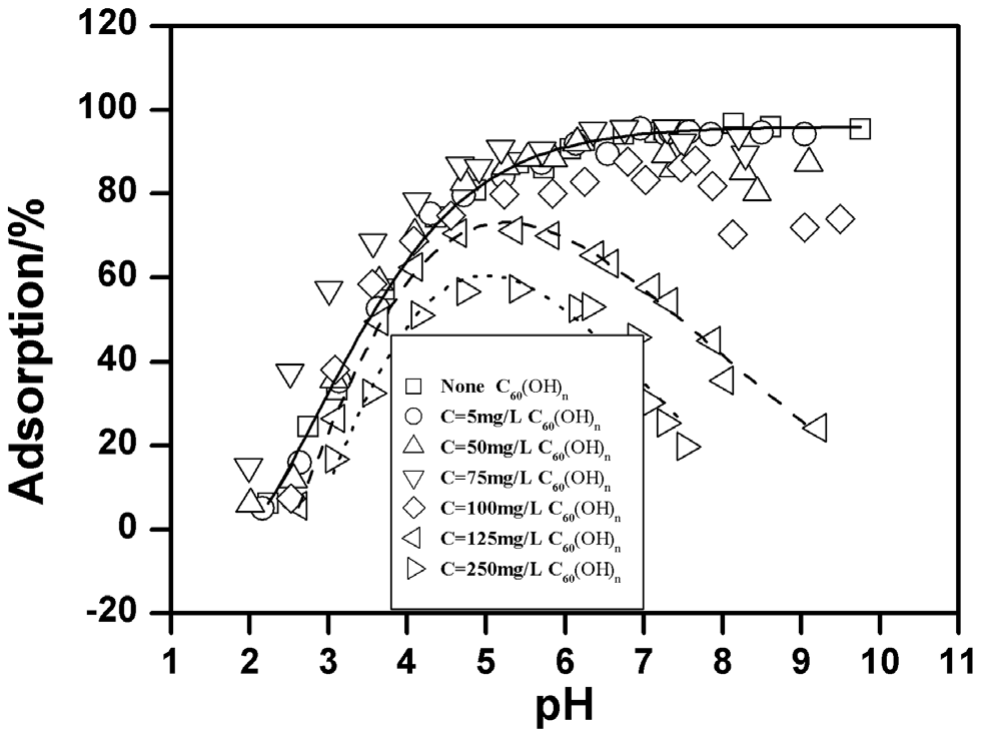
Effect of C_60_(OH)_n_ on Cu(II) adsorption on oMWCNTs as a function of pH, *m/V*  = 0.5 g/L, *T = *25±1°C, *I* = 0.01 mol/L NaCl, C[Cu^2+^]initial  = 1.87×10^−4^ mol/L.

**Figure 9:**
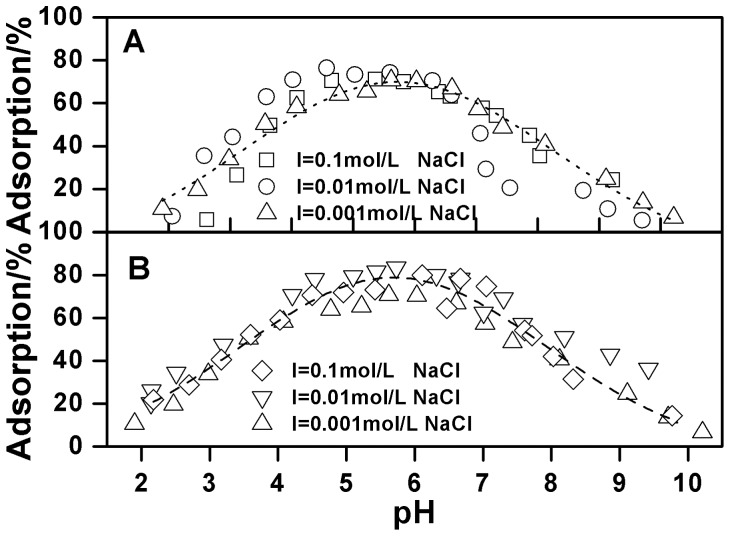
Effect of C_60_(OH)_n_ on Cu(II) adsorption on oMWCNTs as a function of pH at different ionic strength, *m/V*  = 0.5 g/L, *T = *25±1°C, C[Cu^2+^]initial  = 1.87×10^−4^ mol/L, (A) C[C_60_(OH)_n_] = 125 mg/L; (B) C[C_60_(OH)_n_] = 250 mg/L.

The pH-dependent Cu(II) adsorption onto oMWCNTs in the absence and presence of C_60_(OH)_n_ is given in Figure 8. One can see that C_60_(OH)_n_ has almost no effect on Cu(II) adsorption on the surface of oMWCNTs at its lower concentration (i.e. 5 mg/L), but the adsorption of Cu(II) onto oMWCNTs decreases with increasing concentration of C_60_(OH)_n_. When the concentration of C_60_(OH)_n_ is 125 mg/L, the adsorption of Cu(II) onto oMWCNTs increases from approximate 26% at pH 3, to nearly 71% at pH 5.4 and decreases to 50% at pH 7.5. When adding 250 mg/L C_60_(OH)_n_, the adsorption of Cu(II) is lower than when 125 mg/L C_60_(OH)_n_ is added. The adsorption of Cu(II) on oMWCNTs increases from nearly 16% at pH 3, to approximately 57% at pH 5.4 and decreases to 20% at pH 7.5. This indicates that C_60_(OH)_n_ inhibits the adsorption of Cu(II) on oMWCNTs. Nonetheless, it can also be seen from Figure 8 that the adsorption of Cu(II) on the oMWCNTs still increases as pH rises to pH of about 5, but sharply decreases with pH above 5 in the presence of C_60_(OH)_n_. According to the literature, the effect of organic species on metal adsorption on oMWCNTs is mainly attributed to the complexation between organic materials, metal ions, and surface functional groups of oMWCNTs by hydrophobic interaction, electrostatic attraction or repulsion, hydrogen bonding between the −OH and the tube surface −OH or−COOH groups, and π-π interactions between the phenolics and the carbon nanotubes [20], [55]. Generally, organic materials act as a “bridge” between metal ions and oMWCNTs to enhance the ability of oMWCNTs to adsorb Cu(II) from the solution. According to this explanation, if the interaction between C_60_(OH)_n_, metal ions, and oMWCNTs occurs, the adsorption of Cu(II) would increase due to the formation of three component complexation. However, this phenomenon does not appear in Figure 8. Moreover, this deduction does not explain why the addition of Cu(II) and C_60_(OH)_n_ do not affect the adsorption of Cu(II) and C_60_(OH)_n_ onto oMWCNTs (Table S2 in File S1). Consequently, it is possible to exclude the potential role of C_60_(OH)_n_ between Cu(II) and oMWCNTs. Two other possible reasons for the inhibition of C_60_(OH)_n_ are as follows: (a) there is an interaction between C_60_(OH)_n_ and Cu(II), and C_60_(OH)_n_ is not adsorbed to oMWCNT surfaces, so that more Cu(II) is dissolved in solution, thus reducing the adsorption of Cu(II) on the oMWCNTs; (b) C_60_(OH)_n_ is adsorbed onto oMWCNT surfaces, but it does not complex with Cu(II), because the competition of C_60_(OH)_n_ and Cu(II) for adsorption sites of oMWCNTs surface, and the adsorbed Cu(II) on oMWCNTs is “squeezed” down by the adsorbed C_60_(OH)_n_, thus weakening the adsorption of Cu(II) to oMWCNTs. The first case assumes that C_60_(OH)_n_ is not adsorbed on the surface of oMWCNTs, because the number of hydroxyl groups of C_60_(OH)_n_ are more than that of oMWCNTs, and the complex degree of C_60_(OH)_n_ with Cu(II) is greater than that of oMWCNTs with Cu(II). Therefore, the adsorbed Cu(II) on the oMWCNTs surface is in turn adsorbed to C_60_(OH)_n_. Because of the adsorption competition for the functional group of C_60_(OH)_n_ and oMWCNTs, Cu(II) adsorption is reduced on oMWCNT surfaces. However, the adsorption of Cu(II) on oMWCNTs increases with increasing pH at acid pH values, and the first case cannot reasonably explain the experimental results; in addition, the TEM (Figure 1B) picture indicates that C_60_(OH)_n_ is adsorbed on the surface of oMWCNTs; this is also a contradiction with the first assumption. Therefore, the first explanation can be ruled out. If C_60_(OH)_n_ and Cu(II) connect together due to complexation, the changing trend of Cu(II) on oMWCNTs either rises more than without C_60_(OH)_n_ or declines, but this is inconsistent with the experimental results. Therefore, the second explanation is obviously reasonable since higher concentrations of C_60_(OH)_n_ does not accelerate the Cu(II) adsorption on the oMWCNTs surface. Figure 9 illustrates that the concentration of background electrolyte has no significant affect on the adsorption of Cu(II) on oMWCNTs in the presence of C_60_(OH)_n_. This indicates that C_60_(OH)_n_ does not interact with Na^+^ in the background solution. It also verifies that the interaction between C_60_(OH)_n_ and the metal ions does not occur when adding C_60_(OH)_n_. The presence of π-π stacking interactions are supportive of the sorption of aromatic C_60_(OH)_n_ to the benzene rings of the MWCNT sidewalls. Besides, electron-donating (OH) constituents on C_60_(OH)_n_ can enhance its adsorption rate onto oMWCNTs. The dissociation constants (p*Ka*) of C_60_(OH)_n_ (organic base) and C_60_(C(COOH)_2_)_n_ (organic acid) are shown in Table S1 in File S1. When pH < p*Ka*, the non-dissociated species and the dissociated species are dominated by organic acids and organic bases, respectively; meanwhile, the hydroxyl groups of oMWCNTs carry positive charges due to protonation (pH < pH_pzc_). The deprotonation of functional groups on oMWCNT surfaces decreases with increasing pH due to deprotonation of C_60_(OH)_n_. Thus the adsorption of Cu(II) on oMWCNTs rises slowly with increasing pH due to the competition of H^+^ and Cu^2+^ for the sorption site on oMWCNTs at pH <4.6. More and more negative C_60_(OH)_n_ are adsorbed onto oMWCNTs due to its greater electron donating effect of −O^−^ at pH >4.6, thus resulting in the enhancement of space hindrance effect of oMWCNTs. Additionally, π-π stacking interactions are stronger than chemical complexation, and the increasing adsorption of C_60_(OH)_n_ on oMWCNTs also “squeezes” down Cu(II) adsorption leading to further decline. In short, the adsorption of C_60_(OH)_n_ onto oMWCNTs affects the surface characteristics and sorption sites, resulting in the observed changes of Cu(II) adsorbed on oMWCNTs.

**Figure 10:**
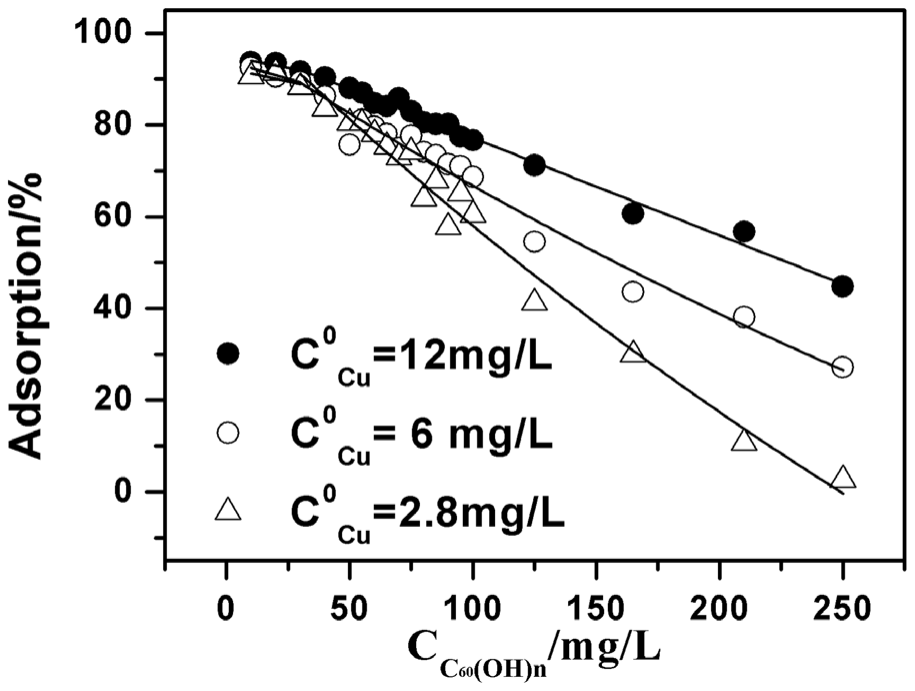
Effect of Cu(II) initial concentrations on Cu(II) adsorption onto oMWCNTs as a function of C_60_(OH)_n_ initial concentrations, *m/V*  = 0.5 g/L, pH = 7.00±0.10, *I* = 0.01 mol/L NaCl, *T = *25±1°C.

**Figure 11:**
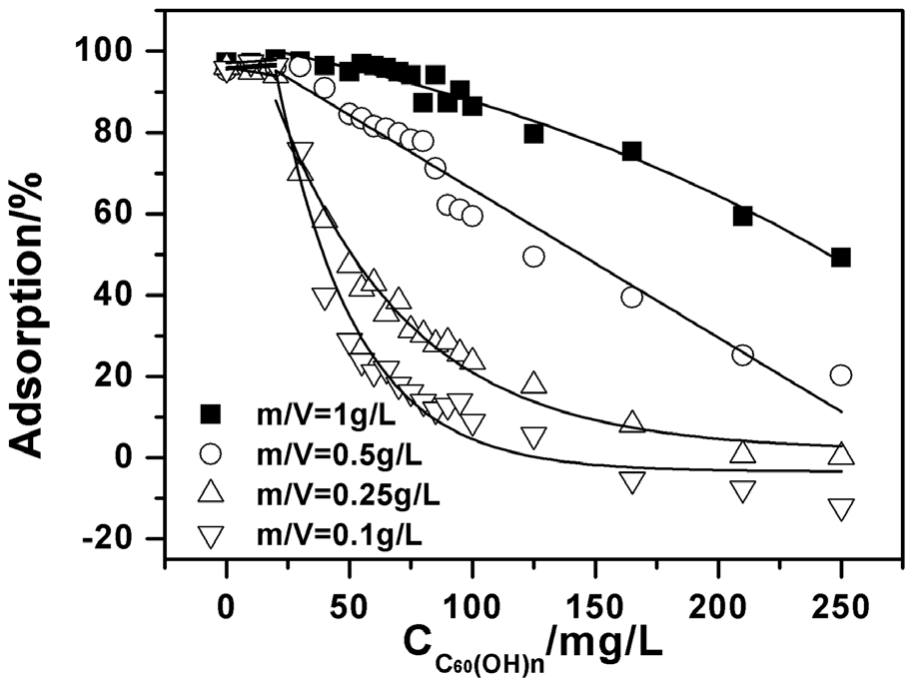
Effect of oMWCNTs dosage on Cu(II) adsorption onto oMWCNTs as a function of C_60_(OH)_n_ initial concentrations, pH = 7.00±0.10, *I* = 0.01 mol/L NaCl, *T = *25±1°C, C[Cu^2+^]initial  = 12 mg/L.

To further investigate the adsorption process of C_60_(OH)_n_ and Cu(II) onto oMWCNTs, the adsorption of Cu(II) onto oMWCNTs as a function of: 1) the initial concentration of C_60_(OH)_n_ at different ratios of solid to liquid; and 2) the initial concentrations of Cu(II), are shown in Figure 10 and Figure 11. As can be seen from Figure 10 and 11, C_60_(OH)_n_ has almost no influence on Cu(II) adsorption at low concentration. Whereas, it starts to inhibit the adsorption ability of oMWCNTs for Cu(II) when the concentration of C_60_(OH)_n_ is higher than 50 mg/L. Figure 10 shows that when the initial concentration of Cu(II) is 1.87×10^−4^ mol/L, its adsorption percentage on the oMWCNTs decreases from about 94% to about 45%; the Cu(II) adsorption decreases from ∼93% to 27% at 9.44×10^−5^ mol/L. When the initial Cu(II) concentration becomes 3.31×10^−5^ mol/L, the adsorption percentage drops from 91% to 3%. In other words, the Cu(II) adsorption percentage decreases regularly as the initial concentration of Cu(II) is reduced. This also shows that the Cu (II) is only adsorbed on oMWCNT surfaces, and has no interaction with C_60_(OH)_n_. Figure 11 illustrates that when the dosage of oMWCNTs are 0.25 g/L and 0.1 g/L and the concentration of C_60_(OH)_n_ is less than 100 mg/L, the adsorption of Cu(II) on oMWCNTs falls sharply. The reduction of Cu(II) adsorption becomes mild when the concentration of C_60_(OH)_n_ is higher than 100 mg/L. This indicates that the low concentration of oMWCNTs does not offer enough available sites for the adsorption of C_60_(OH)_n_, but with the increasing dosage of oMWCNTs, the available adsorption sites on oMWCNTs surfaces gradually increase, leading to more and more C_60_(OH)_n_ adsorption. Consequently, adsorption of Cu(II)on oMWCNTs decreases sharply.

Figure 8 shows that the adsorption of Cu(II)declines rapidly as the pH rises when the concentration of C_60_(OH)_n_ is more than 100 mg/L, whereas C_60_(OH)_n_ suppresses the Cu(II) adsorption after its concentration exceeds a critical value. This indicates that there might be two different C_60_(OH)_n_ binding sites on oMWCNTs surfaces. Carbon nanotubes are composed of hexagonal carbon rings and MWCNTs are mainly comprised of dozens of layers of coaxial tubes. Aromatic C_60_(OH)_n_ could connect with the sidewalls of oMWCNTs through π-π stacking interactions. However, according to the literature [56], the walls of carbon nanotubes are not completely composed of hexatomic rings, wherein some of the hybridized carbon atoms can easily be modified. The π electron mobility of this hybrid ring is lower than that of a complete hexatomic ring. And the π-π stacking interaction capability of the hybrid ring is relatively weak. Therefore, we ascribe the hexatomic ring on the sidewalls of oMWCNTs as the first binding site of C_60_(OH)_n_, also known as the strong adsorption sites. We designate the heterol ring of oMWCNTs as the second binding site, also referred to as the weak adsorption sites. C_60_(OH)_n_ is adsorbed on the first adsorption site of oMWCNTs by the strong π-π interaction at low concentration. The adsorption of Cu(II) mainly occurs with the surface functional groups of oMWCNTs through chemical complexation [57]. The adsorption of C_60_(OH)_n_ and Cu(II) are independent processes. Therefore, C_60_(OH)_n_ has no effect on the Cu(II) adsorption at its low concentration, but C_60_(OH)_n_ begins to occupy the second binding site after the first adsorption sites are saturated as its concentration increases. Then the adsorbed Cu(II) ions are repelled by the space steric effect caused by the adsorbed C_60_(OH)_n_, thus the adsorption of Cu(II) begins to decrease.

The following assumptions can be made for modeling purposes: (1) there are two adsorption sites on oMWCNTs, the second binding site is defined as the common action sites for C_60_(OH)_n_ and Cu(II) sorption; (2) the adsorption sites are uniformly distributed on oMWCNTs. Only monodendate binding is occurring at the binding sites (i.e., one cation per site).

The initial concentration of C_60_(OH)_n_ is defined as *C_f_* (mg/L); the adsorption saturation concentration of Cu(II) is *C_p_* (mg/L); the adsorption amount of C_60_(OH)_n_ on the second site of oMWCNTs is *Q_f_* (mg/L); the adsorption reduction of Cu(II) on the second site is *Q_c_* (mg/L); the adsorption percentage of Cu(II) on oMWCNTs are as follows: 
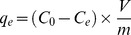
(4)

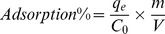
(5)Where *C_o_* (mg/L) is the initial concentration of Cu(II) before adsorption; *q_e_* (mg/g) represents the equilibrium concentration of Cu(II) in the solid phase after adsorption; *C_e_* (mg/L) is the equilibrium concentration of Cu(II) in the liquid phase.

When *C_e_* < *C_p_*, the adsorption of Cu(II) and C_60_(OH)_n_ are independent, the adsorption of Cu(II) does not vary with changes of C_60_(OH)_n_. Therefore, 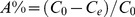
(6)
*C_e_* > *C_p_*, indicates that the adsorbed Cu(II) is transferred to the solution from the surface of oMWCNTs. *C_e_* has a relationship with the adsorption of C_60_(OH)_n_. According to the assumptions mentioned above, the increasing rate of adsorption of C_60_(OH)_n_ on the second adsorption sites on oMWCNTs is Δ*Q_f_*/Δ*t* (because the adsorption rate of C_60_(OH)_n_ is related to its initial concentration, we denote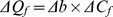; where Δ*b* is the parameter for characterization of the adsorption of C_60_(OH)_n_, it depends on the species and surface character of C_60_(OH)_n_ and the nature of oMWCNTs. Meanwhile, the adsorption of Cu(II) is reduced with the adsorption of C_60_(OH)_n_. Then the decreasing rate of Cu(II) is −Δ*Q_c_*/Δ*t*. The increasing rate of C_60_(OH)_n_ adsorption on oMWCNTs is equal to the decreasing rate of Cu(II) adsorption. As illustrated in Figure 10, the decreasing rate of Cu(II) is slower with higher concentration. This means the squeezed capacity of C_60_(OH)_n_ for the adsorbed Cu(II) is relatively weaker at the high concentration of Cu(II). When the adsorption of C_60_(OH)_n_ and the reduction of Cu(II) reaches equilibrium, it can be expressed by the following expressions:



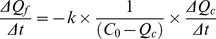
(7)

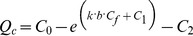
(8)


Therefore,
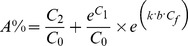
(9)


When 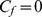,
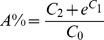
(10)


When 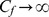, 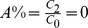


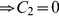
(11)


Substituting *C_2_*  =  0 to the above formulation (10), so:
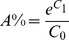


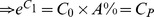


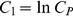
(12)


Given the above, 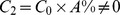; and together with 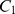 are the performance parameters for the sorption characterization of oMWCNTs. These parameters depend on the surface character of oMWCNTs and the nature and species of Cu(II); *k* represents the rate constant. We define this model as the double sorption site model (DSSM). Figure 10 and 11 presents the fitting curve based on the DSSM formulation. The fitting results are shown in Table 4. The R^2^ value shows that the adsorption isotherm of Cu(II) on oMWCNTs as a function of the initial concentration of C_60_(OH)_n_ can be confidently simulated with the DSSM model.

**Table 4:** Parameters of double adsorption site model.

	double adsorption site model			
m/V(g·L^−1^)	C_2_/C_0_	e^c1^/C_0_	k·b	R^2^
1	128.23	−25.71	−220.12	0.9698
0.5	1.12×10^7^	−1.12×10^7^	–	0.9523
0.25	1.72	125.31	53.38	0.9786
0.1	−3.39	184.26	31.84	0.9555

	double adsorption site model			
C_0_(mg·L^−1^)	C_2_/C_0_	e^c1^/C_0_	k·b	R^2^
12	3.29	−3.29	−1.55	0.9885
6	−115.59	215.25	602.01	0.9705
2.9	−186.85	293.53	551.47	0.9805

### Influence of C_60_(C(COOH)_2_)_n_ on the adsorption of Cu(II)

**Figure 12:**
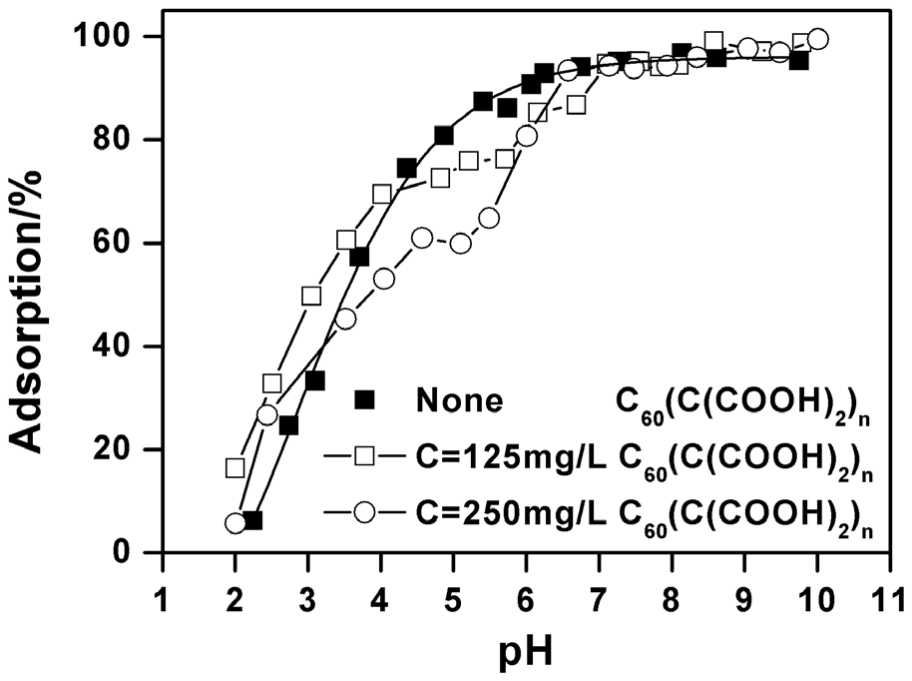
Effect of C_60_(C(COOH)_2_)_2_ on Cu(II) adsorption onto oMWCNTs as a function of pH, *m/V*  = 0.5 g/L, *T = *25±1°C, *I* = 0.01 mol/L NaCl, C[Cu^2+^]initial  = 1.87×10^–4^ mol/L.

Figure 12 illustrates the adsorption of Cu(II) on oMWCNTs as a function pH in the presence of C_60_(C(COOH)_2_)_n_. The adsorption of Cu(II) declines at pH 4–6 due to the inhibition of C_60_(C(COOH)_2_)_n_. Subsequently, Cu(II) adsorption gradually increases to a maximum and then remains stable. C_60_(C(COOH)_2_)_n_ can be excluded from the interaction between C_60_(C(COOH)_2_)_n_ and Cu(II) because there is no increasing adsorption of Cu(II). However, the adsorption mechanism of the combined impact of C_60_(C(COOH)_2_)_n_ and C_60_(OH)_n_ behaves differently. Due to the protonation of C_60_(C(COOH)_2_)_n_, the competition between H^+^ and Cu^2+^ for the adsorption site of oMWCNTs results in the declining adsorption of Cu(II) at pH 4–6. Because of the electron withdrawing effect of the carboxyl group on C_60_(C(COOH)_2_)_n_, the π-π stacking interactions of C_60_(C(COOH)_2_)_n_ with the oMWCNTs benzene ring are weakened. Moreover, the steric hindered reaction of C_60_(C(COOH)_2_)_n_ is stronger than that of C_60_(OH)_n_, and C_60_(C(COOH)_2_)_n_ cannot easily be adsorbed onto oMWCNTs like C_60_(OH)_n_. This may be the reason why the TEM photos of the dispersion of oMWCNTs and C_60_(C(COOH)_2_)_n_ have not shown C_60_(C(COOH)_2_)_n_ connected to oMWCNT surfaces. Therefore, the increasing space hindrance effect of C_60_(C(COOH)_2_)_n_ reduces the C_60_(C(COOH)_2_)_n_ adsorption on oMWCNTs surfaces, and consequently Cu(II) adsorption on oMWCNTs gradually rises and then maintains steady state at pH >6.

**Figure 13:**
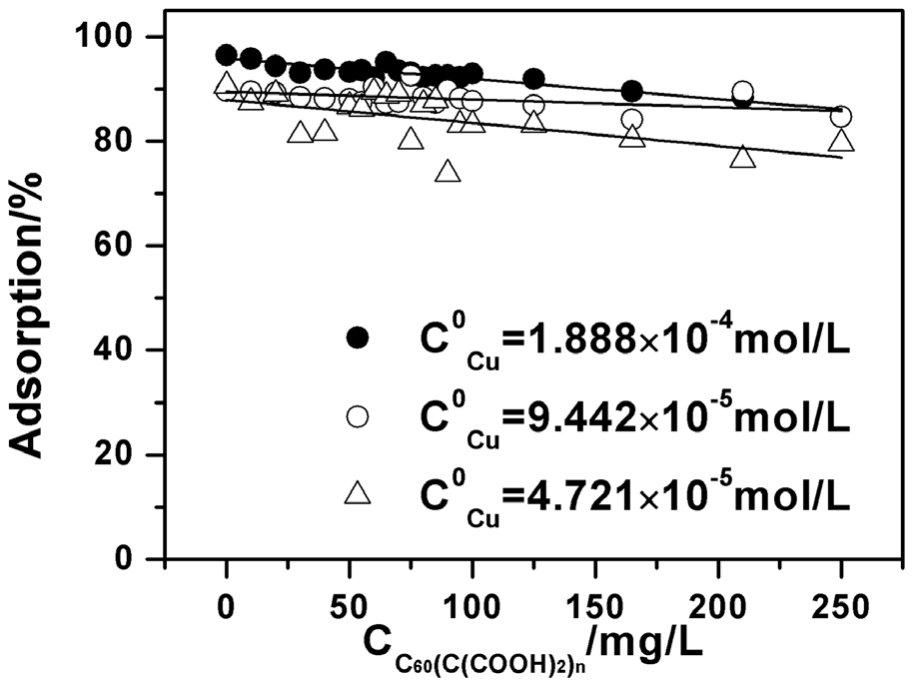
Effect of Cu(II) initial concentrations on Cu(II) adsorption onto oMWCNTs as a function of C_60_(C(COOH)_2_)_n_ initial concentrations, *m/V*  = 0.5 g/L, pH = 5.50±0.10, *I* = 0.01 mol/L NaCl, *T = *25±1°C.

**Figure 14:**
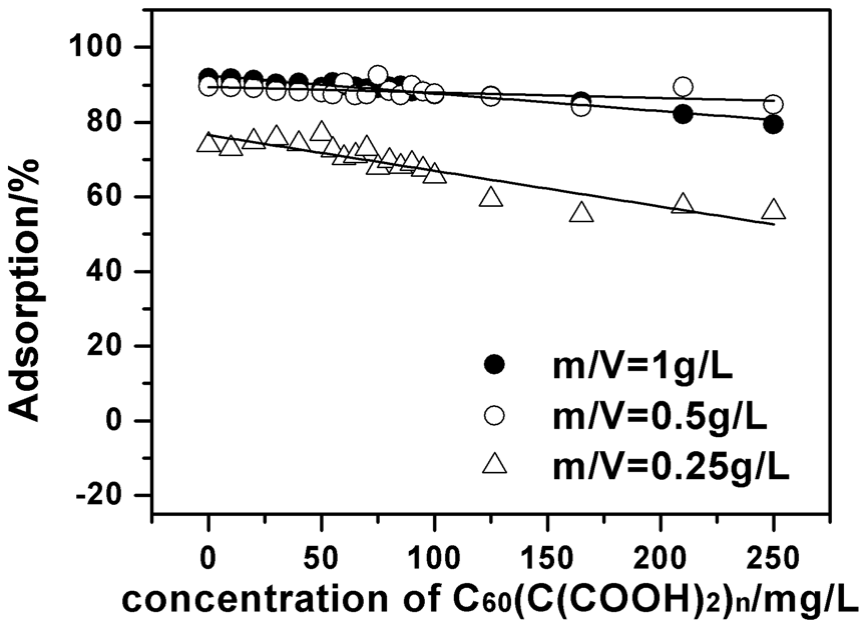
Effect of oMWCNTs dosage on Cu(II) adsorption onto oMWCNTs as a function of C_60_(C(COOH)_2_)_n_ initial concentrations, pH = 5.50±0.10, *I* = 0.01 mol/L NaCl, *T = *25±1°C, C[Cu^2+^]initial  = 1.87×10^–4^mol/L.

Likewise, the influence of C_60_(C(COOH)_2_)_n_ on Cu(II) adsorption onto oMWCNTs are evaluated at different initial concentrations of Cu(II) and concentration of oMWCNTs at pH 5.5±0.05. The results are shown in Figure 13 and Figure 14. The adsorbed Cu(II) reduces slightly with the increasing concentration of C_60_(C(COOH)_2_)_n_. This shows that the inhibition mode of C_60_(C(COOH)_2_)_n_ and C_60_(OH)_n_ are essentially different. Although C_60_(C(COOH)_2_)_n_ and C_60_(OH)_n_ can be adsorbed to the sidewalls of oMWCNTs by the π-π interaction, the species and the connecting styles of surface functional groups introduced on C_60_ vary from each other, leading to declining Cu(II) adsorption on the surface of oMWCNTs to varying degrees.

## Conclusions

The results of this work indicate that the adsorption of Cu(II)onto oMWCNTs is strongly pH dependent and weakly ion strength dependent. Chemical complexation is the main mechanism of Cu(II) adsorption to oMWCNTs. The negative effects of C_60_(OH)_n_ and C_60_(C(COOH)_2_)_n_ on the adsorption of Cu(II) on oMWCNTs are completely different. The DSSM model simulates the adsorption isotherms of Cu(II) adsorption as a function of initial concentration of C_60_(OH)_n_. These results are important in understanding the transport of Cu(II) from two kinds of carbon nanomaterials and their behavior in the environment.

## Supporting Information

File S1 Figure S1, FTIR spectrum of (A) oMWCNTs; (B) oMWCNTs+Cu(II); (C) oMWCNTs+Cu(II)+C_60_(OH)_n_; (D) oMWCNTs+Cu(II)+C_60_(C(COOH)_2_)_n_. Figure S2, Relative proportion of Cu(II) species as a function of pH; C[Cu^2+^]initial  = 1.87×10^−4^ mol·L^−1^, *I* = 0.01 mol/L NaCl, *T = *25±1°C, *P_CO2_* = 10^−3.58^ atm. Figure S3, Effect of C_60_(OH)_n_ on the zeta potential of oMWCNTs. Figure S4, Effect of C_60_(C(COOH)_2_)_n_ on the zeta potential of oMWCNTs. Figure S5, The dispersibility of oMWCNTs after adding different concentration soluble fullerene: (A) single oMWCNTs; (B) oMWCNTs +10 mg C_60_(C(COOH)_2_)_n_; (C) oMWCNTs +100 mg C_60_(C(COOH)_2_)_n_; (D) oMWCNTs +1000 mg C_60_(C(COOH)_2_)_n_; (E) oMWCNTs +10 mg C_60_(OH)_n_; (F) oMWCNTs + 100 mg C_60_(OH)_n_; (G) oMWCNTs + 1000 mg C_60_(OH)_n_. Table S1, The parameters of C_60_(C(COOH)_2_)_n_ and C_60_(OH)_n_ species. Table S2, The influence of adding order of species on Cu((II) sorption on oMWCNTs. Table S3, Constants for the kinetic adsorption of Cu(II) on oMWCNTs using different adsorption models.
(DOCX)

## References

[1] Heath JR (1999) Nanoscale materials. Accounts of Chemical Research 32: 388–388.

[2] Davoren M, Herzog E, Casey A, Cottineau B, Chambers G, et al (2007) In vitro toxicity evaluation of single walled carbon nanotubes on human A549 lung cells. Toxicology in Vitro 21: 438–448. 17125965 10.1016/j.tiv.2006.10.007

[3] Hyung H, Fortner JD, Hughes JB, Kim J-H (2006) Natural Organic Matter Stabilizes Carbon Nanotubes in the Aqueous Phase. Environmental Science & Technology 41: 179–184. 10.1021/es061817g 17265945

[4] Abbaspour A, Izadyar A (2007) Carbon nanotube composite coated platinum electrode for detection of Cr(III) in real samples. Talanta 71: 887–892. 19071390 10.1016/j.talanta.2006.05.085

[5] Sitko R, Zawisza B, Malicka E (2012) Modification of carbon nanotubes for preconcentration, separation and determination of trace-metal ions. TrAC Trends in Analytical Chemistry 37: 22–31.

[6] Pyrzynska K (2010) Carbon nanostructures for separation, preconcentration and speciation of metal ions. TrAC Trends in Analytical Chemistry 29: 718–727.

[7] Chao-Yin K (2009) Water purification of removal aqueous copper (II) by as-grown and modified multi-walled carbon nanotubes. Desalination 249: 781–785.

[8] Pyrzyńska K, Bystrzejewski M (2010) Comparative study of heavy metal ions sorption onto activated carbon, carbon nanotubes, and carbon-encapsulated magnetic nanoparticles. Colloids and Surfaces A: Physicochemical and Engineering Aspects 362: 102–109.

[9] Gao Z, Bandosz TJ, Zhao Z, Han M, Qiu J (2009) Investigation of factors affecting adsorption of transition metals on oxidized carbon nanotubes. Journal of Hazardous Materials 167: 357–365. 19264402 10.1016/j.jhazmat.2009.01.050

[10] Li Y, Liu F, Xia B, Du Q, Zhang P, et al (2010) Removal of copper from aqueous solution by carbon nanotube/calcium alginate composites. Journal of Hazardous Materials 177: 876–880. 20083351 10.1016/j.jhazmat.2009.12.114

[11] Bystrzejewski M, Pyrzyńska K (2011) Kinetics of copper ions sorption onto activated carbon, carbon nanotubes and carbon-encapsulated magnetic nanoparticles. Colloids and Surfaces A: Physicochemical and Engineering Aspects 377: 402–408.

[12] Wu C-H (2007) Studies of the equilibrium and thermodynamics of the adsorption of Cu^2+^ onto as-produced and modified carbon nanotubes. Journal of Colloid and Interface Science 311: 338–346. 17442333 10.1016/j.jcis.2007.02.077

[13] Li Y-H, Ding J, Luan Z, Di Z, Zhu Y, et al (2003) Competitive adsorption of Pb^2+^, Cu^2+^ and Cd^2+^ ions from aqueous solutions by multiwalled carbon nanotubes. Carbon 41: 2787–2792.

[14] Rao GP, Lu C, Su F (2007) Sorption of divalent metal ions from aqueous solution by carbon nanotubes: A review. Separation and Purification Technology 58: 224–231.

[15] Pyrzynska K (2008) Carbon Nanotubes as a New Solid-Phase Extraction Material for Removal and Enrichment of Organic Pollutants in Water. Separation & Purification Reviews 37: 372–389.

[16] Hyung H, Kim J-H (2008) Natural Organic Matter (NOM) Adsorption to Multi-Walled Carbon Nanotubes: Effect of NOM Characteristics and Water Quality Parameters. Environmental Science & Technology 42: 4416–4421. 18605564 10.1021/es702916h

[17] Chen GC, Shan X, Wang YS, Pei Z, Shen XE, et al (2008) Effects of copper, lead, and cadmium on the sorption and desorption of atrazine onto and from carbon nanotubes. Environmental Science & Technology 42: 8297–8302. 19068809 10.1021/es801376w

[18] Chakrapani N, Zhang YM, Nayak SK, Moore JA, Carroll DL, et al (2003) Chemisorption of Acetone on Carbon Nanotubes. The Journal of Physical Chemistry B 107: 9308–9311.

[19] Sheng G, Li J, Shao D, Hu J, Chen C, et al (2010) Adsorption of copper(II) on multiwalled carbon nanotubes in the absence and presence of humic or fulvic acids. Journal of Hazardous Materials 178: 333–340. 20153111 10.1016/j.jhazmat.2010.01.084

[20] Tan X, Fang M, Chen C, Yu S, Wang X (2008) Counterion effects of nickel and sodium dodecylbenzene sulfonate adsorption to multiwalled carbon nanotubes in aqueous solution. Carbon 46: 1741–1750.

[21] Jia Z, Wang Z, Xu C, Liang J, Wei B, et al (1999) Study on poly(methyl methacrylate)/carbon nanotube composites. Materials Science and Engineering: A 271: 395–400.

[22] Balasubramanian K, Burghard M (2005) Chemically Functionalized Carbon Nanotubes. Small 1: 180–192. 17193428 10.1002/smll.200400118

[23] Hussain CM, Saridara C, Mitra S (2008) Microtrapping characteristics of single and multi-walled carbon nanotubes. Journal of Chromatography A 1185: 161–166. 18282580 10.1016/j.chroma.2008.01.073

[24] Hirsch A, Chen Z, Jiao H (2000) Spherical Aromaticity in Ih Symmetrical Fullerenes: The 2(N+1)2 Rule. Angewandte Chemie International Edition 39: 3915–3917. 10.1002/1521-3773(20001103)39:21<3915::AID-ANIE3915>3.0.CO;2-O 29711706

[25] Tagmatarchis N, Shinohara H (2001) Fullerenes in medicinal chemistry and their biological applications. Mini Rev Med Chem 1: 339–348. 12369961 10.2174/1389557013406684

[26] Chawla P, Chawla V, Maheshwari R, Saraf SA, Saraf SK (2010) Fullerenes: from carbon to nanomedicine. Mini Rev Med Chem 10: 662–677. 20236059 10.2174/138955710791572497

[27] Sardenberg RB, Teixeira CE, Pinheiro M, Figueiredo JM (2011) Nonlinear conductivity of fullerenol aqueous solutions. ACS Nano 5: 2681–2686. 21388223 10.1021/nn102913p

[28] Bühl M, Hirsch A (2001) Spherical aromaticity of fullerenes. Chem Rev 101: 1153–1183. 11710216 10.1021/cr990332q

[29] Montes-Morán MA, Suárez D, Menéndez JA, Fuente E (2004) On the nature of basic sites on carbon surfaces: an overview. Carbon 42: 1219–1225.

[30] Li T, Li X, Huang K, Jiang H, Li J (1999) Synthesis and characterization of hydroxylated fullerene epoxide—an intermediate for forming fullerol. Journal of Central South University of Technology 6: 35–36.

[31] Ye C, Chen C, Chen Z, Meng H, Xing L, et al (2006) In situ observation of C_60_(C(COOH)_2_)_2_ interacting with living cells using fluorescence microscopy. Chinese Science Bulletin 51: 1060–1064.

[32] Avilés F, Cauich-Rodríguez JV, Moo-Tah L, May-Pat A, Vargas-Coronado R (2009) Evaluation of mild acid oxidation treatments for MWCNT functionalization. Carbon 47: 2970–2975.

[33] Lu C, Liu C, Rao GP (2008) Comparisons of sorbent cost for the removal of Ni^2+^ from aqueous solution by carbon nanotubes and granular activated carbon. Journal of Hazardous Materials 151: 239–246. 17618049 10.1016/j.jhazmat.2007.05.078

[34] Xu J-Y, Han K, Li S-X, Cheng J-S, Xu G-T, et al (2009) Pulmonary responses to polyhydroxylated fullerenols, C_60_(OH)_x_ . Journal of Applied Toxicology 29: 578–584. 19484703 10.1002/jat.1442

[35] Cheng F, Yang X, Zhu H, Sun J, Liu Y (2000) Synthesis of oligoadducts of malonic acid C_60_ and their scavenging effects on hydroxyl radical. Journal of Physics and Chemistry of Solids 61: 1145–1148.

[36] Pardanaud C, Aréou E, Martin C, Ruffe R, Angot T, et al (2012) Raman micro-spectroscopy as a tool to measure the absorption coefficient and the erosion rate of hydrogenated amorphous carbon films heat-treated under hydrogen bombardment. Diamond and Related Materials 22: 92–95.

[37] Hiura H, Ebbesen TW, Tanigaki K, Takahashi H (1993) Raman studies of carbon nanotubes. Chemical Physics Letters 202: 509–512.

[38] Ferrari A, Robertson J (2001) Resonant Raman spectroscopy of disordered, amorphous, and diamondlike carbon. Physical Review B 64: 075414.

[39] Xu D, Tan X, Chen C, Wang X (2008) Removal of Pb(II) from aqueous solution by oxidized multiwalled carbon nanotubes. Journal of Hazardous Materials 154: 407–416. 18053642 10.1016/j.jhazmat.2007.10.059

[40] Chen C, Li X, Zhao D, Tan X, Wang X (2007) Adsorption kinetic, thermodynamic and desorption studies of Th(IV) on oxidized multi-wall carbon nanotubes. Colloids and Surfaces A: Physicochemical and Engineering Aspects 302: 449–454.

[41] Ho YS (1995) Adsorption of heavy metals from waste streams by peat, Ph.D. Thesis. The University of Birmingham, Birmingham UK.

[42] Hu J, Chen C, Zhu X, Wang X (2009) Removal of chromium from aqueous solution by using oxidized multiwalled carbon nanotubes. Journal of Hazardous Materials 162: 1542–1550. 18650001 10.1016/j.jhazmat.2008.06.058

[43] Alkan M, Doğan M (2001) Adsorption of Copper(II) onto Perlite. Journal of Colloid and Interface Science 243: 280–291. 10.1016/j.jcis.2005.05.027 16023129

[44] Lee SM, Davis AP (2001) Removal of Cu(II) and Cd(II) from aqueous solution by seafood processing waste sludge. Water Research 35: 534–540. 11229008 10.1016/s0043-1354(00)00284-0

[45] Üçer A, Uyanik A, Aygün ŞF (2006) Adsorption of Cu(II), Cd(II), Zn(II), Mn(II) and Fe(III) ions by tannic acid immobilised activated carbon. Separation and Purification Technology 47: 113–118.

[46] Yang S, Li J, Shao D, Hu J, Wang X (2009) Adsorption of Ni(II) on oxidized multi-walled carbon nanotubes: Effect of contact time, pH, foreign ions and PAA. Journal of Hazardous Materials 166: 109–116. 19097690 10.1016/j.jhazmat.2008.11.003

[47] Chen C, Wang X (2006) Adsorption of Ni(II) from Aqueous Solution Using Oxidized Multiwall Carbon Nanotubes. Industrial & Engineering Chemistry Research 45: 9144–9149.

[48] Wang X, Chen C, Hu W, Ding A, Xu D, et al (2005) Sorption of ^243^Am(III) to Multiwall Carbon Nanotubes. Environmental Science & Technology 39: 2856–2860. 15884386 10.1021/es048287d

[49] Lu C, Chiu H (2006) Adsorption of zinc(II) from water with purified carbon nanotubes. Chemical Engineering Science 61: 1138–1145.

[50] Zhao D, Yang X, Zhang H, Chen C, Wang X (2010) Effect of environmental conditions on Pb(II) adsorption on β-MnO_2_ . Chemical Engineering Journal 164: 49–55.

[51] Shi K, Wang X, Guo Z, Wang S, Wu W (2009) Se(IV) sorption on TiO_2_: Sorption kinetics and surface complexation modeling. Colloids and Surfaces A: Physicochemical and Engineering Aspects 349: 90–95.

[52] Hayes K F, Leckie J O (1987) Modeling ionic strength effects on cation adsorption at hydrous oxide/solution interfaces. Journal of Colloid and Interface Science 115: 564–572.

[53] Yavuz Ö, Altunkaynak Y, Güzel F (2003) Removal of copper, nickel, cobalt and manganese from aqueous solution by kaolinite. Water Research 37: 948–952. 12531278 10.1016/s0043-1354(02)00409-8

[54] Wang X, Liang X, Wang Y, Wang X, Liu M, et al (2011) Adsorption of Copper (II) onto activated carbons from sewage sludge by microwave-induced phosphoric acid and zinc chloride activation. Desalination 278: 231–237.

[55] Lin D, Xing B (2008) Adsorption of Phenolic Compounds by Carbon Nanotubes: Role of Aromaticity and Substitution of Hydroxyl Groups. Environmental Science & Technology 42: 7254–7259. 18939555 10.1021/es801297u

[56] Gauden PA, Terzyk AP, Rychlicki G, Kowalczyk P, Lota K, et al (2006) Thermodynamic properties of benzene adsorbed in activated carbons and multi-walled carbon nanotubes. Chemical Physics Letters 421: 409–414.

[57] Di Z-C, Ding J, Peng X-J, Li Y-H, Luan Z-K, et al (2006) Chromium adsorption by aligned carbon nanotubes supported ceria nanoparticles. Chemosphere 62: 861–865. 16403560 10.1016/j.chemosphere.2004.06.044

