# Supplementary material for: Adsorption of Cu(II) on Oxidized Multi-Walled Carbon Nanotubes in the Presence of Hydroxylated and Carboxylated Fullerenes
Source: PLoS One. 2013 Aug 29;8(8):e72475. doi: 10.1371/journal.pone.0072475 (PMC3756995; doi:10.1371/journal.pone.0072475)
Supplement: File S1 — Figure S1, FTIR spectrum of (A) oMWCNTs; (B) oMWCNTs+Cu(II); (C) oMWCNTs+Cu(II)+C60(OH)n; (D) oMWCNTs+Cu(II)+C60(C(COOH)2)n. Figure S2, Relative proportion of Cu(II) species as a function of pH; C[Cu2+]initial = 1.87×10^−4^ mol·L−1, I = 0.01 mol/L NaCl, T = 25±1°C, PCO2 = 10−3.58 atm. Figure S3, Effect of C60(OH)n on the zeta potential of oMWCNTs. Figure S4, Effect of C60(C(COOH)2)n on the zeta potential of oMWCNTs. Figure S5, The dispersibility of oMWCNTs after adding different concentration soluble fullerene: (A) single oMWCNTs; (B) oMWCNTs +10 mg C60(C(COOH)2)n; (C) oMWCNTs +100 mg C60(C(COOH)2)n; (D) oMWCNTs +1000 mg C60(C(COOH)2)n; (E) oMWCNTs +10 mg C60(OH)n; (F) oMWCNTs + 100 mg C60(OH)n; (G) oMWCNTs + 1000 mg C60(OH)n. Table S1, The parameters of C60(C(COOH)2)n and C60(OH)n species. Table S2, The influence of adding order of species on Cu((II) sorption on oMWCNTs. Table S3, Constants for the kinetic adsorption of Cu(II) on oMWCNTs using different adsorption models. (DOCX) [file pone.0072475.s001.docx]

**Supplementary Materials**

For

**Adsorption of Cu(II) on Oxidized Multi-walled Carbon Nanotubes in the presence of Hydroxylated and Carboxylated Fullerenes**

Jing Wang^1^, Zhan Li^2^, Shicheng Li^3^, Wei Qi^1^, Peng Liu^1^, Fuqiang Liu^1^,

Yuanlv Ye^1^, Liansheng Wu^1^, Lei Wang^1^, Wangsuo Wu^1^^[[1]](#footnote-2)^*

*1 Radiochemistry Laboratory, School of Nuclear Science and Technology, Lanzhou University, Lanzhou 730000, PR China*

*2 Institute of Modern Physics, Chinese Academy of Sciences, Lanzhou 730000, PR China*

*3 Institute of Nuclear Physics and Chemistry, China Academy of Engineering Physics, Mianyang 621900, PR China*

Number of pages: 9

Number of figures: 5

Number of tables: 3

**List of Figures**

[Figure S1. FTIR spectrum of (A) oMWCNTs; (B) oMWCNTs+Cu(II);](#_Toc360570484) [(C) oMWCNTs+Cu(II)+C_60_(OH)_n_; (D) oMWCNTs+Cu(II)+C_60_(C(COOH)_2_)_n_. 3](#_Toc360570485)

[Figure S2. Relative proportion of Cu(II) species as a function of pH](#_Toc360570486): [C[Cu^2+^]initial =1.87×10^-4^ mol·L^-1^, *I*=0.01 mol/L NaCl, *T =* 25±1 ºC, *P_CO2_*=10^-3.58^ atm 4](#_Toc360570487)

[Figure S3. Effect of C_60_(OH)_n_ on the zeta potential of oMWCNTs. 5](#_Toc360570488)

[Figure S4. Effect of C_60_(C(COOH)_2_)_n_ on the zeta potential of oMWCNTs. 5](#_Toc360570489)

[Figure S5. The dispersibility of oMWCNTs after adding different concentration soluble fullerene:](#_Toc360570490) [(A) single oMWCNTs; (B) oMWCNTs + 10 mg C_60_(C(COOH)_2_)_n_; (C) oMWCNTs + 100 mg C_60_(C(COOH)_2_)_n_; (D) oMWCNTs + 1000 mg C_60_(C(COOH)_2_)_n_; (E) oMWCNTs + 10 mg C_60_(OH)_n_; (F) oMWCNTs + 100 mg C_60_(OH)_n_; (G) oMWCNTs + 1000 mg C_60_(OH)_n_. 6](#_Toc360570491)

**List of Tables**

[Table S1 the parameters of C_60_(C(COOH)_2_)_n_ and C_60_(OH)_n_ species 7](#_Toc360570492)

[Table S2 the influence of adding order of species on Cu((II) sorption on oMWCNTs 8](#_Toc360570493)

[Table S3 Constants for the kinetic adsorption of Cu(II) on oMWCNTs using different adsorption models 9](#_Toc360570494)

Figure S1. FTIR spectrum of (A) oMWCNTs; (B) oMWCNTs+Cu(II);

(C) oMWCNTs+Cu(II)+C_60_(OH)_n_; (D) oMWCNTs+Cu(II)+C_60_(C(COOH)_2_)_n_.

Figure S2. Relative proportion of Cu(II) species as a function of pH;

C[Cu^2+^]initial =1.87×10^-4^ mol·L^-1^, *I*=0.01 mol/L NaCl, *T =* 25±1 ºC, *P_CO2_*=10^-3.58^ atm.

**Figure S3.** Effect of C_60_(OH)_n_ on the zeta potential of oMWCNTs.

**Figure S4.** Effect of C_60_(C(COOH)_2_)_n_ on the zeta potential of oMWCNTs.


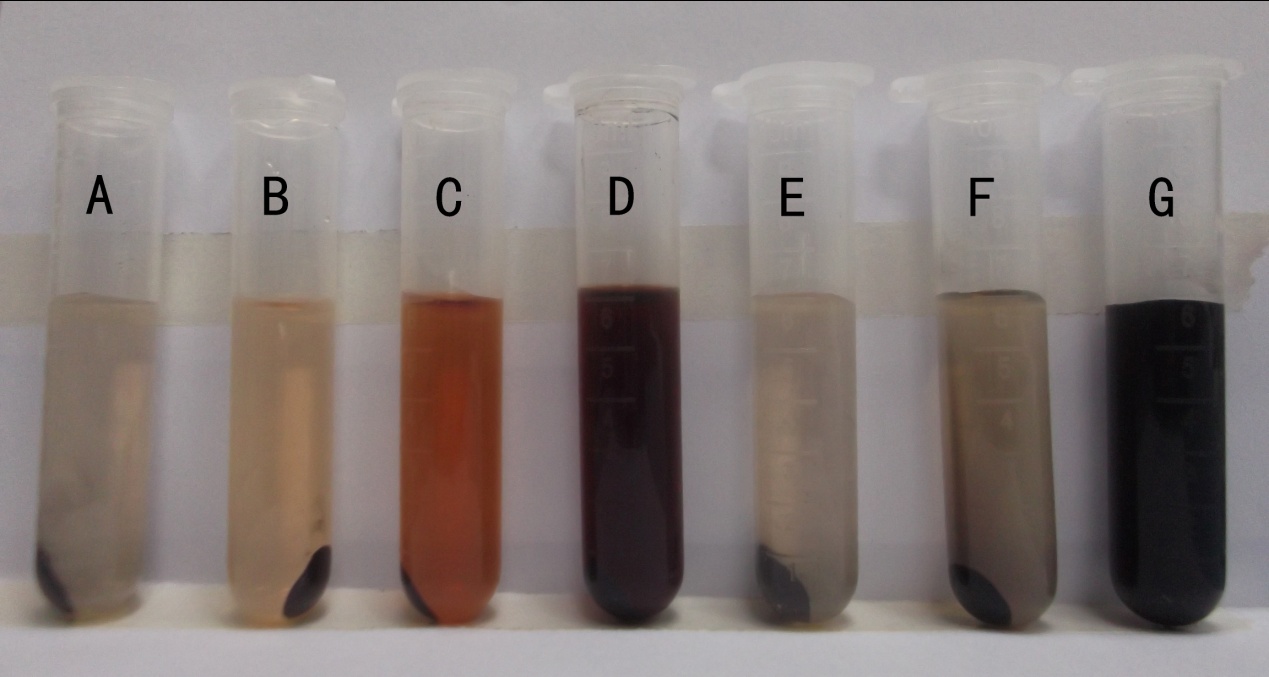


**Figure S5.** The dispersibility of oMWCNTs after adding different concentration soluble fullerene:

(A) single oMWCNTs; (B) oMWCNTs + 10 mg C_60_(C(COOH)_2_)_n_; (C) oMWCNTs + 100 mg C_60_(C(COOH)_2_)_n_; (D) oMWCNTs + 1000 mg C_60_(C(COOH)_2_)_n_; (E) oMWCNTs + 10 mg C_60_(OH)_n_; (F) oMWCNTs + 100 mg C_60_(OH)_n_; (G) oMWCNTs + 1000 mg C_60_(OH)_n_.

**Table S1** the parameters of C_60_(C(COOH)_2_)_n_ and C_60_(OH)_n_ species.

| **n^*^** | **C_60_(OH)_n_** | **C_60_(C(COOH)_n_** |
| --- | --- | --- |
|  | **pK_b1_** | **pK_a1_** |
| 2 | 4.04 | 5.63 |
| 4 | 4.04 | 5.58 |
| 6 | 4.04 | 5.54 |
| 8 | 4.04 | 5.50 |
| 10 | 4.04 | 5.47 |
| 20 | 4.04 | 5.33 |
| 24 | 4.04 | 5.28 |

* the n value according to the literature [[1](#_ENREF_1), [2](#_ENREF_2)].

1. Li, T.; Li, X.; Huang, K.; Jiang, H.; Li, J., Synthesis and characterization of hydroxylated fullerene epoxide—an intermediate for forming fullerol. *J Cent South Univ T* **1999,** *6*, (1), 35-36.

2. Ye, C.; Chen, C.; Chen, Z.; Meng, H.; Xing, L.; Jiang, Y.; Yuan, H.; Xing, G.; Zhao, F.; Zhao, Y.; Chai, Z.; Fang, X.; Han, D.; Chen, L.; Wang, C.; Wei, T., In situ observation of C_60_(C(COOH)_2_)_2_ interacting with living cells using fluorescence microscopy. *Chinese Sci Bull* **2006,** *51*, (9), 1060-1064.

**Table S2** **The influence of adding order of species on Cu((II) sorption on oMWCNTs.**

| **Sequences** | **A** | **Adsorption%** |
| --- | --- | --- |
| oMWCNTs+C_60_(OH)_n_+Cu(II) | 0.533 | 25.19 |
| oMWCNTs+Cu(II)+C_60_(OH)_n_ | 0.486 | 26.45 |
| C_60_(OH)_n_+Cu(II)+oMWCNTs | 0.559 | 21.53 |
| oMWCNTs+C_60_(C(COOH)_2_)_n_+Cu(II) | 0.469 | 28.74 |
| oMWCNTs+Cu(II)+C_60_(C(COOH)_2_)_n_ | 0.494 | 30.66 |
| C_60_(C(COOH)_2_)_n_+Cu(II)+oMWCNTs | 0.474 | 29.16 |

C[Cu^2+^]initial =1.87×10^-4^ mol·L^-1^，*I*=0.01 mol/L NaCl, *T =* 25±1 ºC, *m/V* =0.5 g/L, pH=3.00±0.10.

1. Li, T.; Li, X.; Huang, K.; Jiang, H.; Li, J., Synthesis and characterization of hydroxylated fullerene epoxide—an intermediate for forming fullerol. *J Cent South Univ T* **1999,** *6*, (1), 35-36.

2. Ye, C.; Chen, C.; Chen, Z.; Meng, H.; Xing, L.; Jiang, Y.; Yuan, H.; Xing, G.; Zhao, F.; Zhao, Y.; Chai, Z.; Fang, X.; Han, D.; Chen, L.; Wang, C.; Wei, T., In situ observation of C_60_(C(COOH)_2_)_2_ interacting with living cells using fluorescence microscopy. *Chinese Sci Bull* **2006,** *51*, (9), 1060-1064.

**Table S3** **Constants for the kinetic adsorption of Cu(II) on oMWCNTs using different adsorption models.**

| **Model** | **Function** | **Constant** | **Values** |
| --- | --- | --- | --- |
| First-order rate reaction | ln[1-U(t)]=-k_r_t | k_r_(L/g) | 1.4329 |
|  |  | C_Ae_(mg/L) | 2.8111 |
|  |  | R^2^ | 0.8676 |
| Pseudo-first-order reaction | log(q_e_-q)=log(q_e_)-k_1_t/2.303 | k_1_(L/h) | 1.2215 |
|  |  | q_e_(mg/g) | 5.5981 |
|  |  | R^2^ | 0.8946 |
| Pseudo-second-order reaction | t/q_t_=1/k_2_q_e_^2^+t/q_e_ | k_2_(L/h) | 0.2194 |
|  |  | q_e_(mg/g) | 6.0493 |
|  |  | R^2^ | 0.9992 |
| Intraparticle diffusion rate reaction | q=k_id_t^1/2^ | K_id_(mg/(g·h^1/2^)) | 0.4285 |
|  |  | R^2^ | 0.6247 |

1. *Corresponding author: Wangsuo Wu, Tel & Fax:+8609318913554, E-mail: [wuws@lzu.edu.cn](mailto:wuws@lzu.edu.cn) (W. Wu). [↑](#footnote-ref-2)
